# Mortality and cardiovascular events in diabetes mellitus patients at dialysis initiation treated with glucagon-like peptide-1 receptor agonists

**DOI:** 10.1186/s12933-024-02364-2

**Published:** 2024-07-29

**Authors:** Hsuan-Wen Lai, Chun Yin See, Jui-Yi Chen, Vin-Cent Wu

**Affiliations:** 1 Taipei Medical University, Taipei, Taiwan; 2 Division of Nephrology, Department of Internal Medicine, College of Medicine, National Cheng Kung University Hospital, National Cheng Kung University, Tainan, Taiwan; 3 Graduate Institute of Clinical Medicine, College of Medicine, National Taiwan University, Taipei, 100 Taiwan; 4 Division of Nephrology, Department of Internal Medicine, Chi Mei Medical Centre, Tainan, Taiwan; 5 Department of Health and Nutrition, Chia Nan University of Pharmacy and Science, Tainan, Taiwan; 6 Division of Nephrology, Primary Aldosteronism Centre of Internal Medicine, National Taiwan University Hospital, Taipei, Taiwan; 7 NSARF (National Taiwan University Hospital Study Group of ARF, Consortium for Acute Kidney Injury and Renal Diseases), Taipei, Taiwan; 8 Department of Internal Medicine, National Taiwan University Hospital, 7 Chung-Shan South Road, Taipei, 100 Taiwan

**Keywords:** Renal replacement therapy, Acute kidney injury, GLP-1, Mortality, Major adverse kidney events, Major adverse cardiac events

## Abstract

**Background:**

Glucagon-like Peptide-1 Receptor Agonists (GLP-1RAs) have demonstrated efficacy in improving mortality and cardiovascular (CV) outcomes. However, the impact of GLP-1RAs therapy on cardiorenal outcomes of diabetic patients at the commencement of dialysis remains unexplored.

**Purpose:**

This study aimed to investigate the long-term benefits of GLP-1RAs in type 2 diabetic patients at dialysis commencement.

**Methods:**

A cohort of type 2 diabetic patients initializing dialysis was identified from the TriNetX global database. Patients treated with GLP-1RAs and those treated with long-acting insulin (LAI) were matched by propensity score. We focused on all-cause mortality, four-point major adverse cardiovascular events (4p-MACE), and major adverse kidney events (MAKE).

**Results:**

Among 82,041 type 2 diabetic patients initializing dialysis, 2.1% (*n* = 1685) patients were GLP-1RAs users (mean ages 59.3 years; 55.4% male). 1682 patients were included in the propensity-matched group, treated either with GLP-1RAs or LAI. The main causes of acute dialysis in this study were ischemic heart disease (17.2%), followed by heart failure (13.6%) and sepsis (6.5%). Following a median follow-up of 1.4 years, GLP-1RAs uses at dialysis commencement was associated with a reduced risk of mortality (hazard ratio [HR] = 0.63, *p* < 0.001), 4p-MACE (HR = 0.65, *p* < 0.001), and MAKE (HR = 0.75, *p* < 0.001). This association was particularly notable in long-acting GLP-1RAs users, with higher BMI, lower HbA1c, and those with eGFR > 15 ml/min/1.73m^2^. GLP-1RAs’ new use at dialysis commencement was significantly associated with a lower risk of MACE (*p* = 0.047) and MAKE (*p* = 0.004). Additionally, GLP-1RAs use among those who could discontinue from acute dialysis or long-term RAs users was associated with a lower risk of mortality, 4p-MACE, and MAKE.

**Conclusion:**

Given to the limitations of this observational study, use of GLP-1RAs at the onset of dialysis was associated with a decreased risk of MACE, MAKE, and all-cause mortality. These findings show the lack of harm associated with the use of GLP-1RAs in diabetic patients at the initiation of acute dialysis.

**Supplementary Information:**

The online version contains supplementary material available at 10.1186/s12933-024-02364-2.

## Introduction

According to the International Diabetes Federation (IDF), there are 537 million people worldwide living with type 2 diabetes and it is one of the leading causes of mortality in the twenty first century [1]. The pathophysiology of type 2 diabetes includes polygenic mutations and cardiometabolic risk factors such as obesity. Glucagon-like peptide-1 receptor agonists (GLP-1RAs), the small molecule drugs that mimic the incretin hormones secreted after food ingestion, delay gastric emptying, decrease the surge of postprandial glucose levels and stimulate insulin release from the pancreas [2]. They also promote weight loss by inducing satiety and suppressing appetite and food cravings [3, 4].

GLP-1 receptors are ubiquitously expressed in various organ systems, explains the pleiotropic effects of GLP-1RAs [5]. As new drug applications dictated a greater emphasis on the cardiovascular (CV) outcomes of antidiabetic medications since the year 2008, the cardioprotective properties of GLP-1RAs were also scrutinized [6]. LEADER (Liraglutide Effect and Action in Diabetes: Evaluation of Cardiovascular Outcome Results) trial, which included patients with chronic kidney disease (CKD), which proved the efficacy of liraglutide in reducing the incidence of death from CV causes, nonfatal myocardial infarction (MI) and stroke [7]. Shortly after, SUSTAIN-6 (Trial to Evaluate Cardiovascular and Other Long-Term Outcomes with Semaglutide in Subjects with Type 2 diabetes) proved the noninferiority of GLP-1RAs in reducing major adverse cardiovascular events (MACE) and rates of new or worsening nephropathy [8]. However, these studies typically excluded patients with an estimated glomerular filtration rate (eGFR) of less than 30 ml/min/1.73 m^2^. The 2022 Kidney Disease Improving Global Outcomes (KDIGO) guideline for diabetes management in chronic kidney disease (CKD) recommended GLP-1RAs as prefer antidiabetic therapy for better glycemic control if they are unable to use SGLT2i or metformin [9].

Insulin is often the last resort for patients with advanced CKD and poor glycemic control, while GLP-1RAs have emerged as a feasible alternative for some of these patients in recent years [10]. Nonetheless, only 20–30% of the study population in the GLP-1RAs cardiovascular outcome trials (CVOTs) published from the year 2015 to 2021 had CKD, and patients with advanced kidney disease (eGFR less than 30 ml/min/1.73 m^2^) were even fewer than 3% [7, 8, 11]. Several small-scale studies have suggested the potential benefits of GLP-1RAs for dialysis patients [12]. An exploratory clinical trial of 12 diabetic patients on maintenance dialysis showed that dulaglutide improved glycemic control without increased hypoglycemic episodes [13]. A recent meta-analysis also supported the ability of GLP-1RAs to lower blood glucose in diabetic patients with advanced or end-stage kidney disease (ESKD), however, these patients experienced a higher risk of gastrointestinal (GI) adverse effects such as vomiting. Moreover, the role of GLP-1 in improving CV outcomes and mortality remains inconclusive [14].

The safety and pharmacokinetics of GLP-1RAs in dialysis, potentially affected by kidney function and the dialysis process, remain unclear. A study found no significant changes in plasma liraglutide and glucose levels on dialysis days in patients with type 2 diabetes and ESKD on maintenance hemodialysis [15]. Liraglutide was undetectable in pooled dialysate samples, despite the use of high-flux dialyzers [15]. Recently, in individuals with type 2 diabetes and CKD, the FLOW trial indicates less CKD progression and a reduction in kidney and CV mortality risk [16].

TriNetX serves as a worldwide collaborative health research network platform, offering real-time access to electronic medical records and data sourced from diverse healthcare organizations (HCOs) engaged in real-world practices [17]. TriNetX database comprises healthcare information from over 250 million de-identified patients. In the context of this study, we harnessed the global capabilities of the TriNetX platform to analysis whether the use of GLP-1RAs and clinical outcomes specifically among patients at dialysis commencement.

## Methods

### Study protocol and patients’ selection

TriNetX database, which was recognized as a global health collaborative platform for clinical research, served as the source of data utilized in this study. The broad spectrum of information in this dataset included patient demographics, diagnoses (according to ICD-10-CM codes), procedures (based on ICD-10-PCS or Current Procedural Terminology), medications (coded according to the Veterans Affairs National Formulary), laboratory tests (based on Logical Observation Identifiers Names and Codes), genomics (coded according to the Human Genome Variation Society), and healthcare utilization records from 83 healthcare organizations (HCOs), comprising hospitals, primary care units, and specialized facilities. This platform has already been used in several studies due to its integrity [18, 19]. Based on the database, we constructed a cohort comprising over 2.8 million participants within the period from 1 January 2015 to 1 May 2023, to investigated either GLP-1RAs or long-acting insulin (LAI) treatment efficacy for diabetic patients undergoing acute dialysis. This study followed the Strengthening the Reporting of Observational Studies in Epidemiology (STROBE) reporting guideline for cohort studies.

### Ethics statement

The examination of TriNetX-derived data garnered institutional approval from the Institutional Review Board of Chi-Mei Hospital (Approval No: 11202-002), as well as endorsement from the respective institutional review boards of all participating hospitals. Adherence to the stipulations outlined in both the Health Insurance Portability and Accountability Act and the General Data Protection Regulation is vigilantly upheld through the mechanisms embedded within the TriNetX platform. Owing to the platform’s exclusive consolidation of de-identified data summaries and counts, it has been accorded an informed consent waiver by the Western Institutional Review Board [20]. The current investigation meticulously adheres to the ethical tenets articulated in the Declaration of Helsinki.

### Study cohorts

**Fig. 1:**
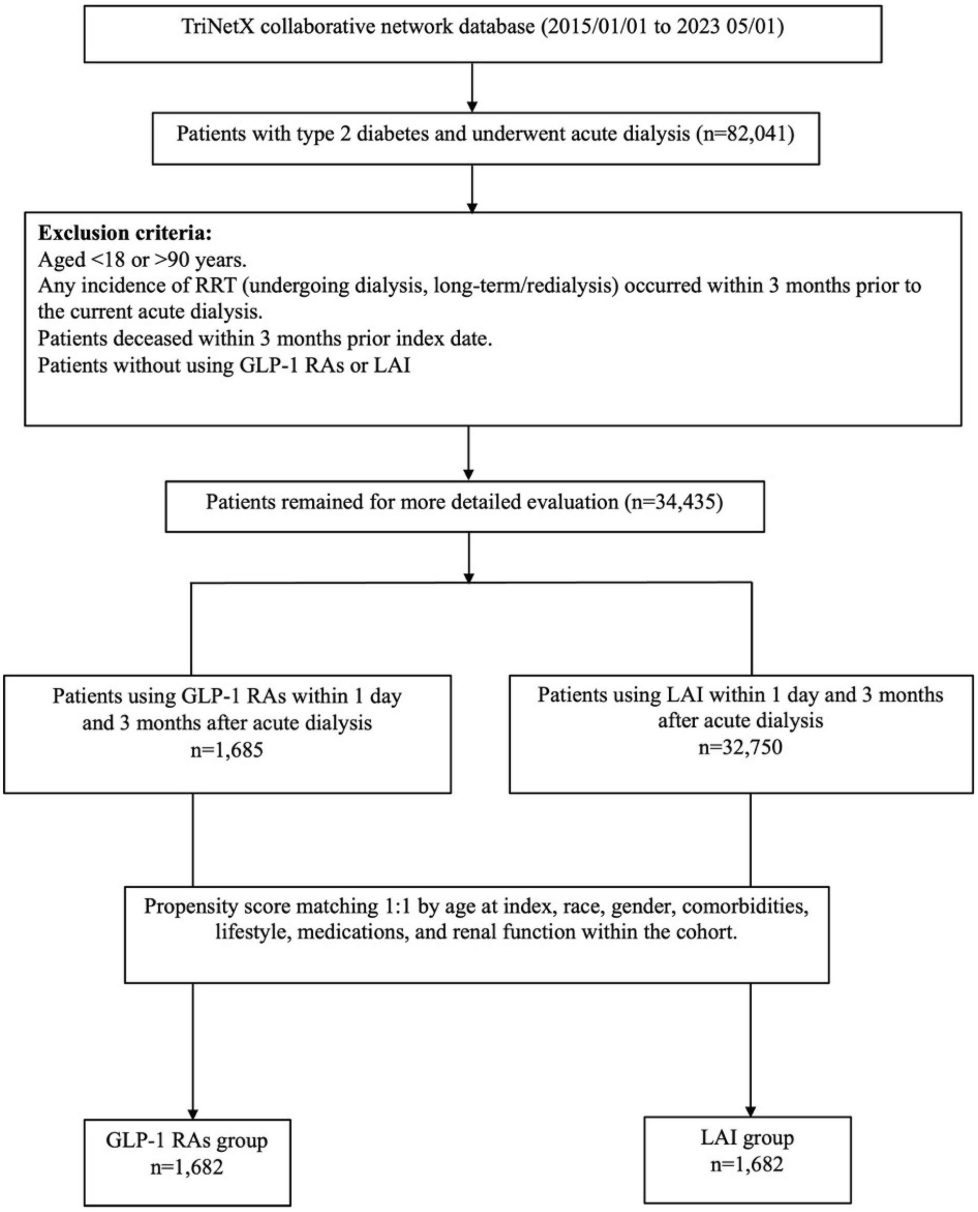
Enrollment algorithm for patients

We identified a cohort of 82,041 patients diagnosed with type 2 diabetes and new-onset dialysis at the enrolled healthcare facilities during the study period (Fig. 1). The index date for all participants was 90 days after the initiation of dialysis. The 3-month window helps mitigate reverse causality effects, ensuring outcomes are more attributed to the GLP-1RAs. It also ensures data reliability, as immediate post-discharge records can be inconsistent.

Inclusion criteria were individuals aged 18 to 90 years, with a confirmed diagnosis of type 2 diabetes, and initializing dialysis during their hospital stay. Key exclusion criteria involved patients undergoing dialysis within three months before the commencement of dialysis. To mitigate immortal bias, individuals who had deceased within three months before the index date were also excluded.

Patients were categorized as GLP-1 RA users if they were prescribed a GLP-1 RA at any point within three months after dialysis commencement. Their counterpart was those prescribed LAI at any point within three months at the same timeframe. The cohort was subsequently stratified into two groups: the GLP-1RAs user group (*n* = 1682), and the LAI user group (*n* = 32,750).

### Prespecified subgroup analyses

Subgroup analyses were systematically conducted to scrutinize potential variations in risk associated with the desired outcomes among users of GLP-1RAs. These prespecified analyses took into consideration various factors, including age, hypertension, heart failure, estimated GFR, proteinuria, the enrolled time period, prior history of GLP-1RAs uses before the discharge, enrolment status before or after 2020, utilization of long- or short-acting GLP-1RAs, concurrent usage of other medications for glycemic control, and the use of renin-angiotensin-aldosterone system (RAAS) blockers. GLP-1RAs long-term user was referred to those who had GLP-1RAs treatment chronically before and after the index date. GLP-1RAs ever user was identified as a patient who has ever, at least within 3 months prior to the index date, utilized GLP-1RAs but discontinued the therapy after dialysis commencement. Conversely, a new user was defined as a patient who had never received GLP-1RAs before the index date but initiated its usage thereafter. Free from dialysis was defined as withdrawal from dialysis events within 180 days after the index date, while dialysis-dependent was defined as any presence of dialysis events occurring within 180 days after the index date. Positive/negative outcomes and exposure controls were meticulously delineated to discern the nuanced impact of GLP-1RAs across these specified subgroups.

### Pre-specified outcomes

The primary outcome was mortality, and the secondary outcomes included major adverse cardiac events (MACE) as well as major adverse kidney events (MAKE). We defined MACE as acute MI, coronary artery bypass, cardiac arrest, cerebral infarction, nontraumatic intracerebral hemorrhage, or all-cause mortality, while MAKE was defined as remaining dialysis at 3 years after the index date or all-cause mortality after the index date. All patients were vigilantly monitored for a period of up to 3 years to ascertain the occurrence of the outcomes of interest.

### Covariates

Different covariates were extracted to account for the variables in baseline characteristics between the two groups. Aside from demographic covariates such as age, sex, race, comorbidities, lifestyle, and medication usage were also considered.

To mitigate prospective disparities in baseline characteristics between the two study cohorts, we systematically amalgamated and algorithmically selected a multitude of high-dimensional covariates evaluated within the 12 months preceding the index event. The identification of comorbidities was based on ICD-10-CM codes (Supplementary table). In addition, we concurrently analyzed confounders including body mass index, and systolic blood pressure, as well as laboratory tests which consisted of HbA1c, estimated glomerular filtration rate (eGFR), potassium, total cholesterol, and alanine aminotransferase before dialysis. Short-acting GLP-1RAs were classified as exenatide and lixisenatide, while long-acting agents included liraglutide, dulaglutide, and semaglutide [21].

### Statistical analysis

Variables were presented in either numerical format, accompanied by means and standard deviations, or categorical format, denoted by counts and percentages, contingent upon the characteristics of the covariates. To mitigate potential confounding variables, we leveraged propensity score matching (PSM) to establish comparable groups, systematically pairing each GLP-1 RA user with an LAI user. Adjusted hazard ratios (aHR) were subsequently computed to assess primary and secondary outcomes between the GLP-1RAs user and control groups [22].

This process was facilitated through the integrated feature within TriNetX, utilizing a greedy nearest-neighbor matching approach. The matching algorithm considered factors such as age, gender, race, comorbidities, lifestyle, medications, and laboratory data. The equilibrium of baseline characteristics in the propensity score-matched populations was gauged using absolute standardized mean differences, whereby a value < 0.1 indicated a negligible difference [23]. To circumvent challenges related to multicollinearity, precedence was accorded to continuous variables. Cases with missing data or those lost to follow-up were excluded to ensure data completeness. E-values were calculated for pre-specified outcomes to address the unmeasured confounders [24].

Moreover, we are currently planning a comprehensive subgroup analysis to explore potential variations in treatment effects within distinct subgroups. This endeavor aims to provide more tailored healthcare recommendations, particularly focusing on patients who have either ever or never used GLP-1RAs before initiating dialysis, as well as those who were able to discontinue dialysis due to the resolution of AKI.

To address concerns regarding immortal or ascertainment bias, our series of analyses involved initiating the timeframe on the 14th, 30th, or 60th days post-acute dialysis. Importantly, post-dialysis mortality was not excluded from the cohort and was followed from the first day after acute dialysis as part of the intention-to-treat (ITT) analysis. We computed E-values using the methodology proposed by Vander Weele and Ding [25]. These values serve as a measure to assess the magnitude of the risk ratio required for unmeasured confounding variables to potentially explain the observed disparities between GLP-1 RA users and non-users regarding the outcomes of interest [26].

To mitigate the impact of reverse causality, the observation period commenced from the index date and was extended for a maximum of 3 years. The relationship between GLP-1RAs users and control groups concerning both primary and secondary outcomes was assessed using the Cox proportional hazards model, allowing for the computation of adjusted hazard ratios (aHRs) [22]. Robust standard errors were employed to account for dependence within matched pairs. The assumption of proportional hazards was scrutinized using the generalized Schoenfeld approach on the TriNetX platform. In instances where assumptions were not met, aHRs were determined for specific time frames. Survival probabilities were computed using the Kaplan-Meier method.

Each analysis included a 95% confidence interval, with statistical significance established at a 2-sided P-value < 0.05.

## Results

### Study population characteristics

Stratification of the expansive study cohort (*n* = 82,041) involved allocating individuals to either the GLP-1RAs group (*n* = 1685, 2.1%) or the LAI control group (*n* = 32,750), based on the administration of GLP-1RAs or LAI within timeframe spanning of one day to three months subsequent to the index date at dialysis commencement. ** (**Table 1**)**. Among the types, dulaglutide (*n* = 715, 42.4%) was the most commonly used GLP-1RAs, followed by semaglutide (*n* = 420, 24.9%) and liraglutide (*n* = 371, 22.0%). Adhering to this methodological rigor, 1,682 GLP-1 RA users and an equivalent number of LAI users, intricately balanced on pertinent covariates, were purposefully selected for the study. The mean age of the GLP-1RAs group was younger than that of the LAI group (59.3 ± 11.6 vs. 61.8 ± 12.8 years, *p* < 0.001) ** (**Table 1**)**. Males were the major gender, as well as the White was the predominant race in both groups. The eGFRs in the GLP-1RAs were higher than the LAI group (41.4 ± 36.1 vs. 36.6 ± 37.1 ml/min/1.73m^2^, *p* < 0.001). After further investigating for the presumptive causes of dialysis commencement, the leading cause was ischemic heart disease (17.2%), followed by heart failure (13.6%) and sepsis (6.5%), with a majority of patients (25.0%) whose baseline kidney function was less than 15 ml/min/1.73m^2^ (Supplementary Table 1).

**Table 1:** Baseline characteristics of study subjects before and after propensity score matching

	Before matched			After matched		
	GLP-1RAs group (*n* = 1685)	LAI group (*n* = 32,750)	ASMD	GLP-1RAs group (*n* = 1682)	LAI group (*n* = 1682)	ASMD
Demographic						
Age	59.3 ± 11.6	61.8 ± 12.8	0.207	59.3 ± 11.6	59.3 ± 12.8	0.002
Male 933 (55.4%)		18,589 (56.8%)	0.028	931 (55.4%)	959 (57.0%)	0.034
White	744 (44.2%)	13,269 (40.5%)	0.074	743 (44.2%)	751 (44.6%)	0.010
Comorbidities, n (%)						
Ischemic heart diseases	585 (34.7%)	9706 (26.6%)	0.109	584 (34.7%)	554 (32.9%)	0.038
Cerebrovascular diseases	214 (12.7%)	3808 (11.6%)	0.033	214 (12.7%)	233 (13.9%)	0.033
Peripheral artery diseases	163 (9.7%)	2389 (7.3%)	0.085	163 (9.7%)	140 (8.3%)	0.048
Heart failure	453 (26.9%)	7765 (23.7%)	0.029	453 (26.9%)	449 (26.7%)	0.005
Atrial fibrillation	185 (11.0%)	3308 (10.1%)	0.029	185 (11.0%)	202 (12.0%)	0.028
Liver cirrhosis	12 (0.7%)	328 (1.0%)	0.031	12 (0.7%)	15 (0.6%)	0.014
Asthma	126 (7.5%)	1638 (5.0%)	0.104	125 (7.5%)	115 (6.9%)	0.026
Neoplasms	305 (18.1%)	5535 (17.0%)	0.032	304 (18.1%)	283 (16.8%)	0.033
Depression	221 (13.1%)	3137 (9.6%)	0.113	221 (13.2%)	193 (11.5%)	0.051
Lifestyle, n (%)						
Nicotine dependence	134 (7.8%)	2279 (7.0%)	0.032	131 (7.8%)	128 (7.6%)	0.007
Alcohol-related disorders	32 (1.9%)	835 (2.5%)	0.043	32 (1.9%)	41 (2.5%)	0.037
Medication, n (%)						
Short acting insulin	769 (45.6%)	12,586 (38.4%)	0.146	767 (45.6%)	857 (51.0%)	0.107
OADs						
Sulfonylureas	241 (14.3%)	2618 (8.0%)	0.201	238 (14.1%)	228 (13.6%)	0.017
Thiazolidinedione	110 (6.5%)	651 (2.0%)	0.226	107 (6.4%)	102 (6.1%)	0.012
Acarbose	27 (1.6%)	295 (0.9%)	0.062	27 (1.6%)	19 (1.1%)	0.041
DPP4i	207 (12.3%)	2568 (7.8%)	0.148	206 (12.2%)	204 (12.1%)	0.004
SGLT2i	175 (10.4%)	695 (2.1%)	0.347	175 (10.4%)	70 (4.1%)	0.243
Antihypertensive medications						
ACEI or ARB	730 (43.3%)	9099 (27.8%)	0.329	728 (43.3%)	729 (43.3%)	0.001
Beta blockers	954 (56.6%)	14,246 (43.5%)	0.264	952 (56.6%)	969 (57.6%)	0.021
CCB	823 (48.9%)	11,871 (36.2%)	0.257	822 (48.9%)	693 (47.1%)	0.034
Diuretics	917 (54.4%)	13,116 (40.1%)	0.291	917 (54.5%)	881 (52.4%)	0.042
Aspirin	674 (40.0%)	9607 (29.3%)	0.225	672 (40.0%)	679 (40.4%)	0.009
Clopidogrel	246 (14.6%)	3341 (10.2%)	0.134	246 (14.6%)	246 (14.6%)	< 0.001
Statins	1,047 (62.1%)	12,536 (38.3%)	0.491	1,044 (62.1%)	1075 (63.9%)	0.038
Febuxostat	38 (2.3%)	385 (1.2%)	0.085	38 (2.3%)	23 (1.4%)	0.067
Laboratory						
Potassium [mmol/L]	4.3 ± 0.6	4.3 ± 0.7	0.035	4.3 ± 0.6	4.3 ± 0.7	0.035
Cholesterol [mg/dL]	154 ± 51.8	154 ± 57.0	0.002	154 ± 51.8	152 ± 64.5	0.021
eGFR [mL/min/1.73m2]						
≥ 30	704 (41.8%)	11,770 (35.9%)	0.120	501 (35.1%)	498 (34.9%)	0.007
<30	795 (47.2%)	15,331 (46.8%)	0.007	715 (50.1%)	722 (50.6%)	0.026
HbA1C						
≥ 7%	731 (43.4%)	8765 (26.8%)	0.354	728 (43.3%)	738 (43.9%)	0.012
< 7%	473 (28.1%)	7808 (23.8%)	0.097	470 (27.9%)	503 (29.9%)	0.043
BMI						
≥ 30 kg/m2	470 (27.9%)	5331 (16.3%)	0.283	470 (27.9%)	458 (27.2%)	0.016
< 30 kg/m2	271 (16.1%)	5171 (15.8%)	0.008	271 (16.1%)	271 (16.1%)	< 0.001
SBP [mmHg]	131 ± 23.9	131 ± 28.3	0.014	131 ± 24	131 ± 28.4	0.006

*ACEI* angiotensin-converting enzyme inhibitors, *ALT*Alanine aminotransferase, *ASMD* absolute standardized mean differences, *AST* Aspartate aminotransferase, *ARB* angiotensin-receptor blockers, *BMI* body mass index, *CCB* calcium channel blocker, *COPD* chronic obstructive pulmonary disease, *DPP4i* Dipeptidyl peptidase-4 inhibitor, *eGFR* estimated glomerular filtration rate, *GLP-1RAs* glucagon-like peptide-1 receptor agonists, *LAI* long-acting insulin, *SBP* systolic blood pressure, *SD* standard deviation, *SGLT2i* sodium–glucose cotransporter 2 inhibitor

**Fig. 2:**
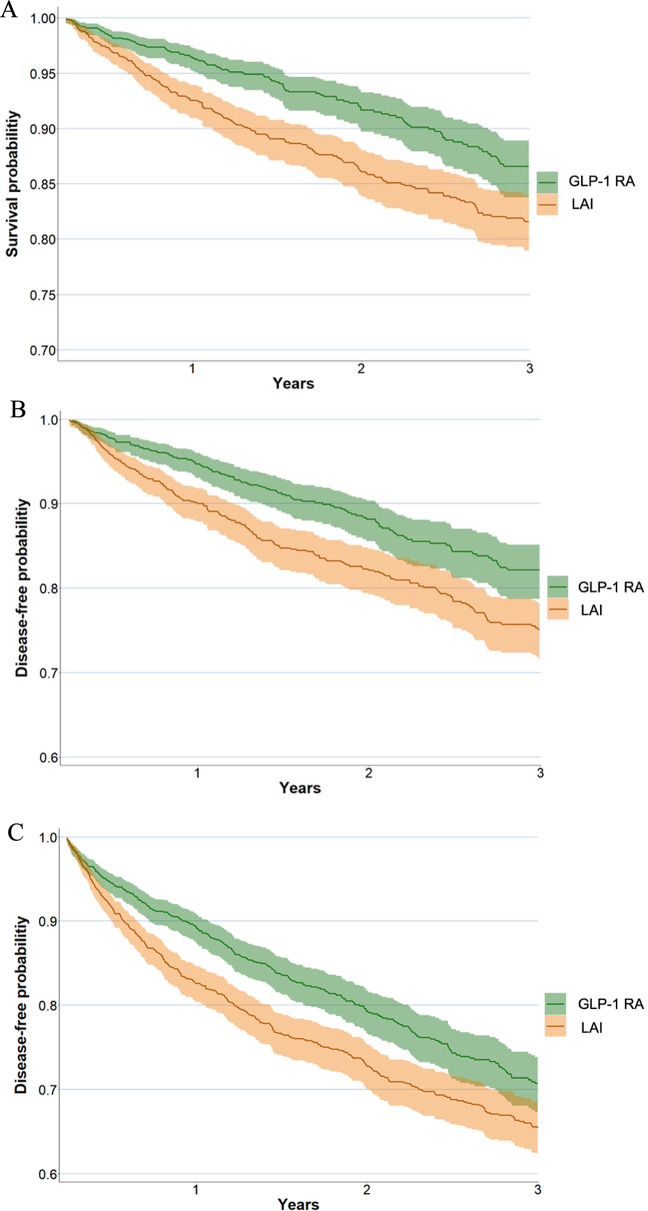
Kaplan–Meier curves of the pre-specified long-term outcome. The green curve represents individuals who are GLP-1 RA users, while the brown curve represents those who are GLP-1 RA non-users (LAI). **A** All-cause mortality (log-rank *p* < 0.001). **B** 4P-MACE (log-rank *p* < 0.001). **C** MAKE (log-rank *p* < 0.001). *GLP-1 RA* glucagon‐like peptide‐1 receptor agonists, *LAI* long‐acting insulin, *4P-MACE* four-point major adverse cardiovascular event, *MAKE* major adverse kidney event

The overall cohort had a median duration of 1.4 years, bracketed by the 25th percentile at 0.8 years and the 75th at 2.1 years, with the 90th percentile reaching 2.6 years (Supplementary Table 2). The all-cause mortality rate was 6.5% in the GLP-1RAs group and 11.0% in the LAI group, where the GLP‐1RAs group users displayed a significantly diminished risk of all-cause mortality (aHR = 0.63, *p* < 0.001) (Table 2, Supplemental Table 3). The E-value for the effect of GLP-1RAs on mortality was 2.54 (with a lower bound of 95% CI of 3.41). Additionally, a decreased risk of MACE (aHR = 0.65, *p* < 0.001), as well as a lower risk of MAKE (aHR = 0.75, *p* < 0.001) were detected in the GLP‐1RAs group compared to the LAI group (Fig. 2, Supplemental Tables 4, 5). The E-value for the effect of GLP-1RAs on MACE was 2.47 (with a lower bound of 95% CI of 3.33), and on MAKE was 1.75 (with a lower bound of 95% CI of 2.07) (Table 2).

**Table 2:** Incidence of outcomes among GLP-1 RA group compared to LAI control group after prosperity score matching

Outcome	Patients with outcome		aHR (95%CI)	Log rank *p*-value	E value for HR	E value for lower bound of 95% CI of HR
	GLP-1RAs group	LAI group				
Primary outcome						
Mortality	6.5% (110/1682)	11.0% (185/1682)	0.63 (0.50–0.80)	< 0.001	2.54	3.41
Secondary outcome						
4P-MACE	9.2% (115/1253)	14.8% (182/1229)	0.65 (0.51–0.82)	< 0.001	2.47	3.33
MAKE	16.1% (270/1682)	21.6% (364/1682)	0.75 (0.64–0.87)	< 0.001	1.75	2.07

*aHR* adjusted hazard ratio, *GLP-1RAs* glucagon-like peptide-1 receptor agonists, *LAI* long-acting insulin, *4P-MACE* four-point major adverse cardiovascular event, *MAKE* major adverse kidney event

### Subgroup analysis

**Fig. 3:**
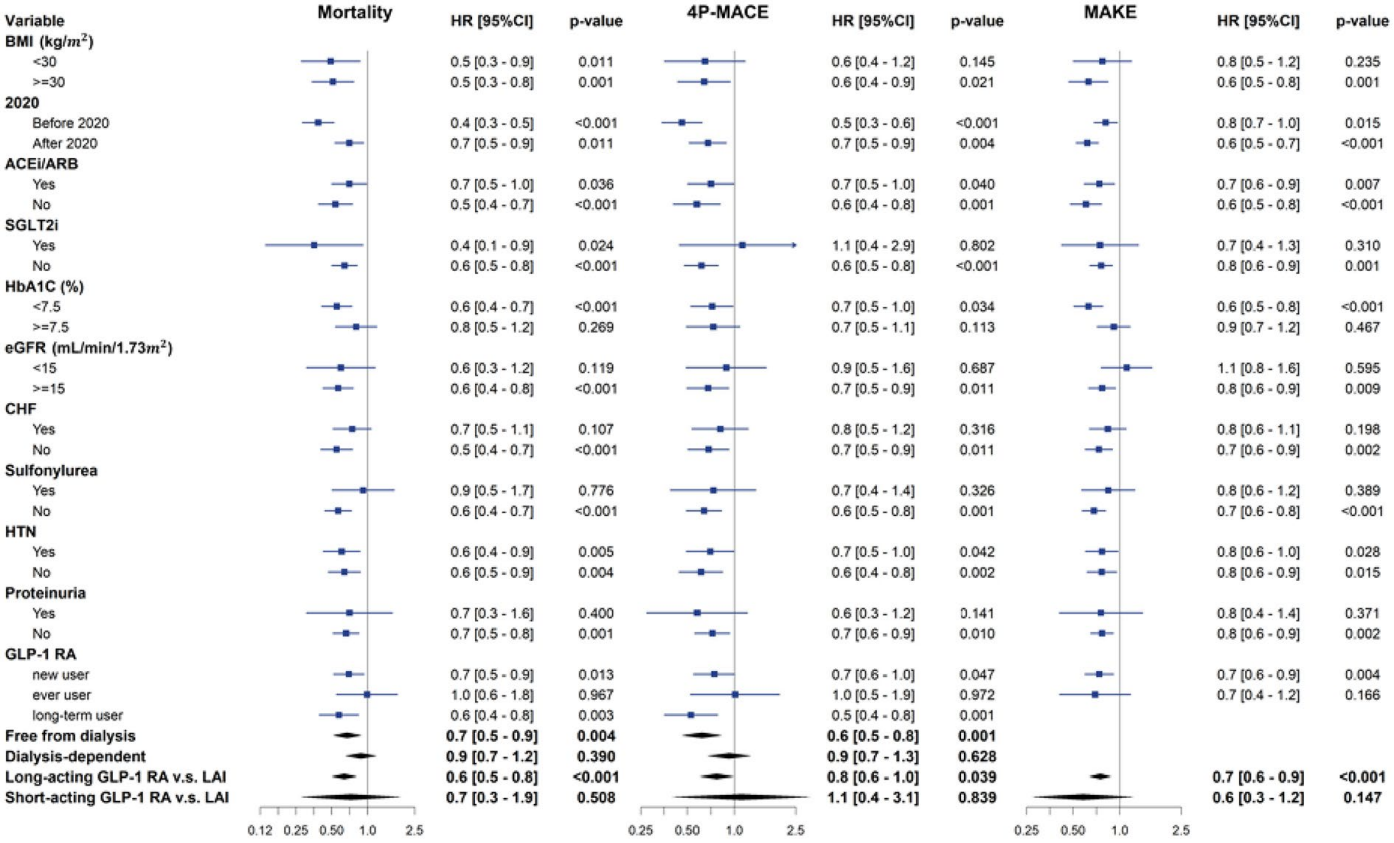
The forest plots illustrated the adjusted HRs of all-cause mortality, 4p-MACE and MAKE for GLP-1 RAs users versus LAI users at dialysis commencement. The plots present both the adjusted HRs and their 95% confidence intervals (CIs), represented as error bars. The vertical line denotes an aHR of 1.00, with lower limits of the 95% CIs exceeding 1.00 indicating a statistically significant increased risk. *ACEI* angiotensin-converting enzyme inhibitors, *ARB* angiotensin-receptor blockers, *BMI* body mass index, *CHF* congestive heart failure, *eGFR* estimated glomerular filtration rate, *GLP‐1 RAs* glucagon‐like peptide‐1 receptor agonists, *HTN* hypertension, *LAI* long‐acting insulin, *4P-MACE* four-point major adverse cardiovascular event, *MAKE* major adverse kidney event, *SGLT2i* sodium–glucose cotransporter 2 inhibitor

Subgroup analyses were undertaken based on medication usage, comorbidities, body mass index, HbA1c, eGFR, and the utilization of short-acting and long-acting GLP-1RAs (Fig. 3). The results consistently unveiled an association between the use of GLP-1RAs and a reduced risk of mortality, MACE and MAKE, which is particularly noteworthy in patients devoid of proteinuria (aHR = 0.66, *p* < 0.001), characterized by HbA1C < 7.5% (aHR = 0.55, *p* < 0.001), and exhibiting BMI ≧ 30 kg/m^2^ (aHR = 0.51, *p* < 0.001). Remarkably, the salutary impact of GLP-1RAs was accentuated in the context of long-acting GLP-1RAs. However, the association between GLP-1RA use and a decreased risk of mortality and MACEs remained consistent, regardless of different timeframes.

Patients with baseline kidney function better than 15 ml/min/1.73m^2^ obtained significant benefited from GLP-1RAs usage compared to the LAI group [mortality (aHR = 0.57, *p* < 0.001), MACE (aHR = 0.68, *p* = 0.011), and MAKE (aHR = 0.77, *p* = 0.009)], but the benefit was not found in those with kidney function less than 15 ml/min/1.73m^2^ [mortality (aHR = 0.60, *p* = 0.119), MACE (aHR = 0.89, *p* = 0.687), and MAKE (aHR = 1.11, *p* = 0.595)]. Among patients who had GLP-1RAs usage before the index date, the trend was only noted in long-term users [mortality (aHR = 0.57, *p* = 0.003), MACE (aHR = 0.53, *p* = 0.001) and MAKE (aHR = 0.69, *p* = 0.003)]. It is noteworthy that GLP-1RAs new users also exhibited a significantly decreased risk of mortality (aHR = 0.70, *p* = 0.013), MACE (aHR = 0.74, *p* = 0.047) and MAKE (aHR = 0.74, *p* = 0.004) compared to the LAI group. Surprisingly, GLP-1RAs ever users who discontinued the treatment did not show any improvement in mortality (aHR = 0.99, *p* = 0.967), MACE (aHR = 1.01, *p* = 0.971) or MAKE (aHR = 1.00, *p* = 0.995).

GLP-1RAs with an exendin backbone are primarily cleared by the kidneys [27]. Our analysis of this subgroup of GLP-1RAs users, including exenatide did not reveal a significant reduction in mortality (adjusted hazard ratio [aHR] = 0.45, *p* = 0.154), major adverse cardiovascular events (MACE) (aHR = 0.48, *p* = 0.193), or major adverse kidney events (MAKE) (aHR = 0.47, *p* = 0.230) compared to the LAI group (Supplementary Table 6).

### Sensitivity and specificity analysis

Angiotensin-converting enzyme inhibitor (ACEI) or angiotensin II receptor blocker (ARB) use among diabetic patients recovered from dialysis-requiring AKI was associated with a lower risk of ESKD and all-cause mortality [28]. As an extension to the current evidence, a positive outcome analysis was performed and showed a significant reduction of MACE and all-cause mortality among ACEI/ARB users in our study population (Supplementary Table 7, Supplementary Fig. 1).

In order to gain insights into the possibility of discontinuing dialysis, compared to LAI, GLP-1RAs were consistently associated with a decreased risk of all-cause mortality, MACE, and MAKE at various selection periods of 14, 30, 60, and 90 days (Supplementary Table 8). To delineate each component of outcomes of interest, we analyzed mortality as well as MI (aHR = 0.63), heart failure (aHR = 0.73), and stroke (aHR = 0.74). The findings indicate that GLP-1RAs use was independently associated with improved outcomes for all these endpoints.

Of note, in dialysis patients who could withdraw from acute dialysis, defined as being free from dialysis at 180 days after the index date (Fig. 3), akin to the main analysis, GLP-1RAs users were associated with a reduced risk of all-cause mortality (aHR = 0.67, *p* = 0.004), and MACE (aHR = 0.62, *p* = 0.001). However, this effect was not observed in patients who continued to be dependent on dialysis.

To investigate the effect of GLP-1RAs on glycemic control, laboratory data of HbA1c were analyzed and showed consistently higher HbA1C levels in the GLP-1 RAs group throughout the study (Supplementary Fig. S2). Additionally, the magnitude of body weight and lipid profile changes among GLP-1RAs and LAI users were analyzed. Though HbA1c and body weight were reduced after treatment, no greater improvement of the lipid profile (low-density lipoprotein, LDL) and systolic blood pressure (SBP) were seen among patients treated with GLP-1RAs compared to LAI (Supplementary Table 9, Figs. S3–S5). Moreover, after dialysis, HbA1C levels and body weight were consistently higher in the GLP-1RAs group compared to the LAI group, indicating that the reduced risk of adverse outcomes associated with GLP-1RAs is not attributable to the “legacy effect”.

We further adjusted for diabetes-related organ injury, such as diabetic retinopathy and neuropathy, as proxies for diabetic severity. The results remained consistent with our main findings [mortality (aHR = 0.56, *p* < 0.001), MACE (aHR = 0.69, *p* = 0.001), and MAKE (aHR = 0.76, *p* < 0.001), Supplementary Table 10], suggesting that the worse outcomes observed in LAI patients were not solely attributable to differences in glycemic control or CVD burden. Additional specificity analyses were performed to compare the efficacy of GLP-1RAs and other second-line antihyperglycemic treatments users (thiazolidinedione (TZD), dipeptidyl peptidase IV inhibitor (DPP4i) or sulfonylureas (SU)), where the results were also consistent with our main finding [mortality (aHR = 0.71, *p* = 0.005), MACE (aHR = 0.76, *p* = 0.028), and MAKE (aHR = 0.84, *p* = 0.031), Supplementary Table 11)].

### Positive and negative outcome controls

We utilized patients with gastroparesis and tachycardia as positive outcome controls to assess potential systematic bias [29, 30]. Notably, the risk of gastroparesis (adjusted hazard ratio [aHR] = 1.36, 95% CI = 1.00–1.85, *p* = 0.047) and tachycardia (aHR = 1.51, 95% CI = 1.00–2.28, *p* = 0.047) was significantly higher in the GLP-1RAs group (Supplementary Fig. 1). Conversely, incidences of melanoma, traumatic head injury, hernia, GI bleeding, and pneumonia, which were not expected to be linked to GLP‐1RAs use, showed no significant differences between the groups. Moreover, the risk of hypoglycemia among type 2 diabetes patients undergoing acute dialysis did not significantly differ between GLP-1RAs users and LAI users (aHR: 1.33 [0.94–1.88], *p* = 0.102) (Supplementary Table 12).

## Discussion

To our knowledge, this is the first and largest cohort study to assess the efficacy of GLP-1RAs in type 2 diabetic patients at dialysis commencement [31]. Our study, based on a worldwide healthcare database, showed that only 2.1% of diabetic patients were given GLP-1RAs at dialysis commencement. Following a median follow-up of 1.4 years, GLP-1RAs use was associated with a 37% reduction of all-cause mortality, 35% reduction of MACE, and 25% reduction of MAKE compared to a matched cohort receiving LAI. Significant associations were observed in long-acting GLP-1RAs users, and those with higher BMI, without proteinuria, lower HbA1c, GLP-1RAs new users, and eGFR > 15 ml/min/1.73 m^2^ before the index date. Patients who could discontinue dialysis exhibited improved mortality and MACE outcomes, although this effect was not observed in those who remained dialysis-dependent or ceased using GLP-1RAs. Further randomized controlled trials are necessary to confirm our results.

### GLP-1RAs were associated with a decrease in mortality

GLP-1RAs use was associated with a greater risk reduction in all-cause mortality compared to LAI, in line with contemporary studies that showed outcome improvement after GLP-1RAs use compared to its counterpart in patients with advanced CKD [32]. Moreover, our analysis revealed that the survival benefits of GLP-1RAs persisted regardless of concurrent use of other anti-diabetic agents or RAAS blockers.

The sustained discrepancy in HbA1C levels prompts consideration of the role of glycemic control in the observed reduction of cardiovascular risk associated with GLP-1RAs therapy use in this population, as indicated in the existing literature [33]. The consistent elevation in HbA1C levels among GLP-1RAs recipients throughout the study, suggests pleotropic effect of GLP-1RAs with additional benefits beyond glucose and weight lowering [33].

A clinical trial [FLOW trial, NCT03819153] on the efficacy of GLP-1RAs in diabetic patients with CKD showed a 25% reduction in kidney disease progression after GLP-1RAs use [16]. We observed a 25% reduction in the need to continue dialysis within 3 years in the GLP-1RAs group.

GLP-1RAs new use was associated with a substantial improvement in all-cause mortality, MACE, and MAKE, but the beneficial effect was not evident in GLP-1RAs ever users who had terminated treatment after dialysis commencement. It is in line with a study that showed an increased likelihood of major CV events after cessation of GLP-1RAs therapy, irrespective of subjects’ prior CV history [34]. This may also imply an enduring protective effect of GLP-1RAs that did not persist beyond the duration of active administration in AKD patients.

Diabetic management in CKD patients is vexatious as glucose homeostasis and drug excretion are altered as the kidney function declines [35]. Insulin is traditionally the last resort for patients with advanced CKD which permits frequent and precise adjustment for better glycemic control [36]. The emergence of long-acting GLP-1RAs offers an alternative to patients at dialysis commencement who had a limited choice of anti-glycemic agent and a higher risk of hypoglycemia [36]. A pilot study showed the superiority of liraglutide in reducing the hyperglycemic period on both dialysis and non-dialysis days [37]. Another study by Yajima et al. showed that dulaglutide as an add-on therapy to insulin could improve blood glucose levels and daily insulin needs without increasing the risk for hypoglycemia [38]. Similarly, our study showed that the risk of hypoglycemia was not different among the GLP-RAs and insulin users at dialysis commencement.

Nonetheless, the conspicuously minimal utilization of GLP-1RAs among these cohorts highlights the imperative for heightened awareness and adoption of this therapeutic regimen in type 2 diabetes. in the associated survival and cardiorenal effects attributed to GLP-1RAs were independent of concurrent administration of other anti-diabetic agents or RAAS blockers. Furthermore, considering the effects observed future inquiries should study the advantages of GLP-1RAs therapy in populations vulnerable to or convalescing from AKI, encompassing individuals undergoing acute dialysis procedures.

### Limitations

Our study has several limitations to consider. Firstly, this was an observational study with propensity score matching which can substantially reduce baseline differences between groups, but it can never correct for unmeasured confounders; therefore, the study cannot determine cause-and-effect relationships. The limited number of patients (2.1%) initiating dialysis within our study cohort impacts the robustness of our findings. The exact rationale for drug prescription, switching of medications, and drug adherence could not be evaluated. However, the study design was intended for treatment and our analysis tried to ensure consistent results, thereby helping to mitigate the potential for guarantee-time bias or immortal time bias [39]. Furthermore, the diseases and medical conditions were identified via the International Classification of Diseases (ICD) codes. Misclassification of the disease codes and underestimation of the prevalence might happen in real-world conditions. However, to try to mitigate these limitations, we employed validated outcome definitions and PSM. Additionally, we utilized various medications as proxies for disease severity, thereby addressing inherent limitations associated with electronic health records. To assess the impact of potential unmeasured confounding, we conducted an E-value analysis, along with propensity score matching and multivariate Cox proportional analysis incorporating various variables. A higher E-value, surpassing our odd ratio, indicates that only a modest unmeasured confounding variable would be needed to neutralize the estimated effect of the covariates.

Of note, the specificity test evaluating hernia, traumatic head injury, melanoma, pneumonia, and GI bleeding revealed no difference between patients with GLP-1RAs users or LAI in our negative control analysis, aiding in the removal of selection bias that can be caused by existing knowledge of an individual’s assignment. Thus, our findings may not apply to patients who are undergoing long-term dialysis. Given these limitations, a prudent interpretation of our results is advisable, and additional research is warranted to confirm and broaden the scope of our observations. To further validate these findings, large randomized controlled trials focusing on this specific patient population are necessary.

## Conclusion

Of note, the specificity test evaluating hernia, traumatic head injury, melanoma, pneumonia, and GI bleeding revealed no difference between patients with GLP-1RAs users or LAI in our negative control analysis, aiding in the removal of selection bias that can be caused by existing knowledge of an individual’s assignment. Thus, our findings may not apply to patients who are undergoing long-term dialysis. Given these limitations, a prudent interpretation of our results is advisable, and additional research is warranted to confirm and broaden the scope of our observations. To further validate these findings, large randomized controlled trials focusing on this specific patient population are necessary.

## Supplementary Material

Below is the link to the electronic supplementary material.

Supplementary Material 1

## Data Availability

No datasets were generated or analysed during the current study.
