# Correction to: Mortality and cardiovascular events in diabetes mellitus patients at dialysis initiation treated with glucagon-like peptide-1 receptor agonists

**DOI:** 10.1186/s12933-024-02452-3

**Published:** 2024-10-14

**Authors:** Hsuan-Wen Lai, Chun Yin See, Jui-Yi Chen, Vin-Cent Wu

**Affiliations:** 1 Taipei Medical University, Taipei, Taiwan; 2 Division of Nephrology, Department of Internal Medicine, College of Medicine, National Cheng Kung University Hospital, National Cheng Kung University, Tainan, Taiwan; 3 Graduate Institute of Clinical Medicine, College of Medicine, National Taiwan University, Taipei, 100 Taiwan; 4 Division of Nephrology, Department of Internal Medicine, Chi Mei Medical Centre, Tainan, Taiwan; 5 Department of Health and Nutrition, Chia Nan University of Pharmacy and Science, Tainan, Taiwan; 6 Division of Nephrology, Primary Aldosteronism Centre of Internal Medicine, National Taiwan University Hospital, Taipei, Taiwan; 7 NSARF (National Taiwan University Hospital Study Group of ARF, Consortium for Acute Kidney Injury and Renal Diseases), Taipei, Taiwan; 8 Department of Internal Medicine, National Taiwan University Hospital, 7 Chung-Shan South Road, Taipei, 100 Taiwan

**Correction: Cardiovascular Diabetology (2024) 23:277** 10.1186/s12933-024-02364-2

In this article Hsuan-Wen Lai was incorrectly denoted as the corresponding author but it should have been Vin-Cent Wu. This has been corrected with this erratum.

The original article has been corrected.

