# Supplementary material for: Mortality and cardiovascular events in diabetes mellitus patients at dialysis initiation treated with glucagon-like peptide-1 receptor agonists
Source: Cardiovasc Diabetol. 2024 Jul 29;23:277. doi: 10.1186/s12933-024-02364-2 (PMC11287940; doi:10.1186/s12933-024-02364-2)
Supplement: Supplementary file 1 — Supplementary Material 1 [file 12933_2024_2364_MOESM1_ESM.docx]

**Supplemental Digital Content to**

**Improvement in Mortality, and Cardiovascular Events in Diabetes Mellitus Patients at Dialysis Initiation with Glucagon-Like Peptide-1 Receptor Agonist**

1. Supplementary material and methods:

　Introduction to the TriNetX database

　　　External validation

　　　Sensitivity analyses, specificity, positive outcome controls, and negative outcome controls

1. Cohorts of type 2 diabetes undergoing acute dialysis who received and not received GLP-1 receptor agonists (GLP-1RAs)
2. Outcome definition
3. Supplementary table

**Suppl. Table 1.** Presumptive causes of AKI

**Suppl. Table 2.** Follow-up period of our analysis for overall cohort

**Suppl. Table 3.** Risk of mortality in type 2 diabetes patients undergoing acute dialysis: comparison between GLP-1RAs users and LAI after propensity score matching

**Suppl. Table 4.** Risk of MACE in type 2 diabetes patients undergoing acute dialysis: comparison between GLP-1RAs users and LAI after propensity score matching

**Suppl. Table 5.** Risk of MAKE in type 2 diabetes patients undergoing acute dialysis: comparison between GLP-1RAs users and LAI after propensity score matching

**Suppl. Table 6.** Risk of all-cause mortality, MACE, and MAKE between exenatide users and LAI in type 2 diabetes patients undergoing acute dialysis
**Suppl. Table 7.** Positive exposure control for all-cause mortality, MACE, and MAKE between ACEI/ARB user and ACEI/ARB non-user in a new user design

**Suppl. Table 8.** Landmark Analysis, Cumulative probability for the risk of all-cause mortality, 4P-MACE, and MAKE within different selective duration of GLP-1RAs
**Suppl. Table 9.** Changes of HbA1c, body weight, low-density lipoprotein (LDL), and systolic blood pressure (SBP) of patients treated with GLP-1RAs and LAI at baseline, day-30-60, and day-60-90

**Suppl. Table 10.** Incidence of outcomes among GLP-1RAs group compared to LAI control group after adjustment for diabetic retinopathy and neuropathy, as proxies for disease severity **Suppl. Table 11.** Risk of all-cause mortality, MACE, and MAKE between GLP-1RAs users and patients using dipeptidyl peptidase-4 inhibitor (DPP4i), thiazolidinedione (TZD) or sulfonylureas in type 2 diabetes patients undergoing acute dialysis **Suppl. Table 12.** Risk of hypoglycemia in type 2 diabetes patients undergoing acute dialysis: between GLP-1RAs users and LAI users after propensity score matching

Supplementary figure

**Suppl. Figure 1.** Positive Outcomes Control, Negative Outcomes Control, and Positive Exposure Control
**Suppl. Figure 2.** Comparison of Effect of Glycemic control between GLP-1 RAs users and LAI group at baseline, day-30-60, and day-60-90
**Suppl. Figure 3.** Changes of body weight of patients treated with GLP-1RA and LAI at baseline, day-30-60, and day-60-90
**Suppl. Figure 4.** Changes of low-density lipoprotein (LDL) of patients treated with GLP-1RA and LAI at baseline, day-30-60, and day-60-90
**Suppl. Figure 5.** Changes of systolic blood pressure (SBP) of patients treated with GLP-1RA and LAI at baseline, day-30-60, and day-60-90
**Suppl. Figure 6.** Graphic abstract

1. STROBE statement checklist
2. Supplementary reference
3. **Supplementary material and methods**

**Introduction to the TriNetX database**

1. **TriNetX federated data network**

TriNetX is the global health research network that connects the world of drug discovery and development from pharmaceutical company to study site, and investigator to patient by sharing real-world data to make clinical and observational research easier and more efficient. TriNetX combines real-time access to longitudinal clinical data with state-of-the-art analytics to optimize protocol design and feasibility, site selection, patient recruitment, and enable discoveries through the generation of real-world evidence. The TriNetX platform is HIPAA and GDPR compliant.

TriNetX was also created to streamline collaborative research between industries and academia in clinical trials. Its goal was to enhance efficiency by allowing researchers to utilize data for designing trials that could potentially meet their accrual needs. Moreover, it aids in pinpointing the best-performing sites for trial openings. Initially, TriNetX reached out to health care organizations with established i2b2 research repositories in 2015 to become data contributors. Over time, TriNetX has improved its data harmonization processes, and replaced i2b2 as the primary patient cohort discovery tool, providing powerful and intuitive query building functions that allow investigators to query EMR data in a completely self-serviceable and secure way. Operating on a hub-and-spoke model, TriNetX involves Pharma and contract research organizations (CROs) sponsors who pay a subscription fee to access aggregate counts from the HCOs within the network. These counts are derived from deidentified patient data. This business approach has proven successful, boasting subscriptions from 14 leading pharmaceutical and CRO sponsors, with a network of 83 healthcare organization data providers.[1]

1. **Network set-up**

TriNetX works as a software-as-a-service platform using Amazon Web Services (AWS) as its underlying architecture, illustrated in Figure 1. Within this setup, the data from Health Care Organizations (HCOs) accessible through TriNetX is stored on an appliance located within each HCO's data center. When onboarding onto the platform, the data gets loaded onto this appliance using an extract-transform-load process, making use of the TriNetX agent's existing capabilities and scripting. Besides i2b2, TriNetX facilitates the incorporation of data from various other source systems by employing a combination of its product features and service capabilities[1].

1. **Security**

TriNetX operates on a secure virtual private cloud adhering to the Health Insurance Portability and Accountability Act (HIPAA) standards, hosted by AWS. This cloud infrastructure complies with various security certifications, including Federal Risk and Authorization Management Program (FedRAMP) and NIST 800-53, in line with industry standards. Access to TriNetX is protected using Transport Layer Security (TLS) and a robust 2048-bit security certificate. The services hosted behind AWS's Elastic Load Balancer adhere to the AWS Elastic Load Balancer Security Policy 2015-05 (details available at <https://aws.amazon.com/security/>).

The TriNetX appliance is designed with a high level of security, with minimal extraneous processes running and stringent access controls. All communication is initiated outbound, and routine penetration and vulnerability tests are conducted to assess the security of the hosted application environment. Expert attestation of the appliance's security is accessible to TriNetX members, accompanied by comprehensive documentation [1]. TriNetX serves as a global federated health research network, offering access to electronic medical records encompassing diagnoses, procedures, medications, laboratory values, and genomic information across extensive healthcare organizations (HCOs). This specific report focuses on a network named Research, comprising 83 healthcare organizations.

1. **Clinical data, controlled terminologies, and semantic mapping**

With a database of over 250 million patients from over 30 countries, the TriNetX platform stands out as an innovative healthcare network that supplies valuable demographic, diagnosis, procedure, medication, and laboratory data tailored for research endeavors. TriNetX has expanded its offerings to encompass tumor registry and molecular genomic data, and recently providing in-depth analyses of current disease prevalence, treatment patterns, and market dynamics based on indication-specific patient-level data [2]. Moreover, there are plans to incorporate vital signs and other observation data relevant to oncology and pulmonology in the imminent future. This expansion aims to furnish researchers with an extensive and real-world dataset of clinical information, fostering evidence-based research initiatives.

The data assimilated by the TriNetX appliance exhibits diversity in origin, contingent on the respective HCO. Some HCOs directly extract data from their electronic health records (EHRs), while others leverage data warehouses with varying common data models, like i2b2 and observational health data sciences and informatics[2]. Commercial EHRs typically employ proprietary code system standards or terminology standards that may differ across countries, such as the United States' Clinical Modification version of the International Classification of Diseases (ICD), Tenth Revision. In the United States, procedures are coded using common terminologies like ICD-10-PCS, Current Procedural Terminology (CPT), whereas other countries lack a universally accepted standard for procedures. Many EHRs include proprietary drug data from sources like First DataBank, Wolters Kluwer’s Medi-Span, and Cerner’s Multum, each utilizing distinct identifiers for the same drug. Medications may also be coded to national drug codes or anatomic therapeutic chemical codes, commonly used in European countries, or local codes. Furthermore, laboratory information systems at HCOs and commercial laboratories rarely adhere to standard codes, like Logical Observation Identifiers Names and Codes (LOINCs), for reporting test results. Through its self-service, HIPAA, GDPR, and LGPD-compliant platform of federated EHR, datasets, and consulting partnerships, TriNetX puts the power of real-world data into the hands of its worldwide community to improve protocol design, streamline trial operations, refine safety signals, and enrich real-world evidence generation.

1. **Data quality**

The challenge of data quality significantly impacts the appropriate utilization of research data, potentially undermining the validity of research outcomes [3]. Despite the widespread adoption of Electronic Health Records (EHRs) driven by federal incentives and meaningful use mandates, the enhancement of data quality within these systems, and consequently for research purposes, remains an ongoing process. Since EHRs primarily serve billing and patient care functions, the data they contain may not consistently meet the rigorous standards required for research endeavors. Addressing this concern necessitates the establishment of a comprehensive framework and methodology for ensuring data quality[4]. Existing research in this domain is often limited, focusing primarily on assessing data quality within individual systems or institutions, with an emphasis on its suitability for clinical care provision[5]. TriNetX has developed a robust methodology for evaluating data quality, characterized by four key components: cleanliness, consistency, correctness, and completeness. This approach encompasses the extraction of data from source systems, subsequent transformation, cleanup, deduplication, de-identification, optional obfuscation, and semantic mapping processes.

1. **Analysis specifications**

The Compare Outcomes Analytic provides four distinct types of analyses: Measure of Association, Survival, Number of Instances, and Lab Result Distribution. The first three analyses offer the functionality to "exclude patients with outcomes prior to the window." This feature proves particularly valuable when examining chronic diseases, as patients who have already experienced the outcome are not susceptible to its development within the specified time frame. When the "exclude patients with outcomes prior to the time window" option remains unchecked, all patients within the cohort are included in the analysis, irrespective of whether they exhibited the outcome before the time window. Conversely, selecting this option results in the exclusion of patients whose medical records indicate the occurrence of the outcome prior to the commencement of the time window. This exclusion encompasses all patients who manifested the outcome before the index event, and any individuals who develop the outcome between the index event and the initiation of the time window will also be excluded if the time window begins subsequent to the index event.

1. **Measure of association analysis**

The Measure of Association Analysis evaluates the proportion of patients experiencing a specified outcome. The resultant summary comprises the count of patients meeting the query criteria in each cohort, the count of patients with the outcome in each cohort, and the risk of the outcome within each cohort. We incorporated adjustments for various factors, including demographic variables (such as age, sex, and race), health conditions (including underlying chronic kidney disease, hyperuricemia, congestive heart failure, ischemic heart diseases, cerebrovascular diseases, overweight, chronic obstructive pulmonary disease, musculoskeletal disease, and malignancy), medication usage (such as Metformin, Sulfonylureas, Acarbose, Insulin, Aspirin, Clopidogrel, Atrovastatin, Allopurinol, Febuxostat, alpha-blocker, beta-blocker, and calcium channel blocker), and clinical metrics (such as body mass index, leukocyte count, platelet count, estimated glomerular rate, proteinuria level, total cholesterol, glycohemoglobin, aspartate transaminase, and B-type natriuretic peptide). Furthermore, the analysis output includes measures such as the Risk Difference (indicating the disparity in risks between cohorts of type 2 diabetes patients underwent acute dialysis who received and did not receive GLP-1 RAs), Risk Ratio (depicting the relative risk in the cohort of GLP-1 RAs users compared to non-users), and Odds Ratio (highlighting the odds ratio in the cohort of GLP-1 RAs users compared to non-users).

1. **Survival analysis**

The Kaplan-Meier Analysis is utilized to estimate the likelihood of the outcome occurring within specific time intervals (in this analysis, daily intervals are utilized). In order to accommodate patients who are no longer part of the cohort during the analysis period, censoring is implemented, which entails the removal of these patients from the analysis following the final recorded event in their medical history. The resulting summary encompasses the count of patients in each cohort (meeting the query criteria), the count of patients experiencing the outcome within the designated time frame, median survival (representing the duration when survival probability falls below 50%; the absence of a value indicates survival does not drop below 50% within the time window), and survival probability at the conclusion of the window (reflecting the percentage of patients surviving at the end of the window). Additionally, the Log-Rank Test, Hazard Ratio, and Proportionality Test are employed in the analysis.

1. **Number of instances analysis**

The Number of Instances Analysis is a methodology used to compute the frequency of an outcome within a specified time frame. This analysis encompasses two configurations: patients with zero instances and the definition of an instance. When opting to exclude patients with zero instances, these individuals are not factored into calculations for mean number of instances, standard deviation, or median. The histogram illustrating the distribution of patients by the number of instances will not feature a bar representing zero instances. Conversely, by choosing to include patients with zero instances, the mean, standard deviation, and median for the number of instances will reflect the entire patient cohort, inclusive of those with zero instances. The histogram will include a bar for zero instances. The determination of an instance influences how the counts are interpreted. When opting for the "Date" setting, each calendar date on which any of the specified outcomes are recorded constitutes one instance. For example, if the outcome comprises "Med A or Med B," and a patient receives "Med A" on January 3, both medications on January 4, and "Med B" on January 6, then the patient is considered to have three instances, corresponding to January 3, January 4, and January 6. It is pertinent to note that if an outcome spans multiple dates, only the initiation date is considered for counting instances. For instance, a patient commencing a hospital stay on January 1, concluding on January 3, initiating another stay on January 10, and concluding on January 15, is regarded as having two instances of the outcome. Opting for "Visit" as an instance counts any visit encompassing the outcome as a single instance, regardless of its recurrence. For instance, if a patient receives an analgesic on each of the three days of an inpatient stay following an index event, these three administrations are treated as one instance since they pertain to the same visit. The output summary includes the patient cohort count, the count of patients within the cohort experiencing the outcome within the specified time window, the mean, standard deviation, and median of the counts, and the median (1+ instances) when patients with zero instances are integrated into the analysis. Additionally, T-Test statistics examining the disparity between the cohorts are provided.

1. **Laboratory results analysis**

In the analysis, solely laboratory results pertinent to the outcomes under examination are incorporated. Moreover, only the most recent laboratory values falling within the specified time window are taken into account. Regarding numeric laboratory results, the outcome summary comprises the count of patients within the cohort meeting the query criteria, the count of patients experiencing the outcome within the designated time frame, the mean, and the standard deviation of the laboratory values within the cohort. Furthermore, T-Test statistics are furnished to assess the disparity between the cohorts. For non-numeric laboratory results, the frequencies of Negatives, Positives, and Unknowns are presented, with these percentages relative to the total counts depicted in a bar chart format.

1. **Limitation**

The TriNetX platform functions based on individual-level data, although researchers do not possess direct access to this specific individual data. Instead, the platform furnishes us with compiled counts and statistical summaries derived from de-identified data, and pseudonymized ID guaranteeing the privacy and confidentiality of the individuals whose data is included. By pooling data from multiple participating institutions, the TriNetX platform, although processing individual-level data internally, restricts researchers' interactions to the analysis of only aggregated and summarized data. This approach is designed to adhere to the guidelines outlined in both the Health Insurance Portability and Accountability Act and the General Data Protection Regulation, thereby maintaining robust standards for data privacy and security.

1. **Cohorts of type 2 diabetes undergoing acute dialysis who received and not received GLP-1RAs**

### Cohort of type 2 diabetes undergoing acute dialysis who received GLP-1RAs

| must have | |  | demographics | | | Age | Age (between 18 and 90 years (most recent occurrence)) |
| --- | --- | --- | --- | --- | --- | --- | --- |
| Group 1A Acute dialysis | | | | | | | |
| must have | | any of | procedure | | | UMLS:CPT:90945 | Dialysis procedure other than hemodialysis (eg, peritoneal dialysis, hemofiltration, or other continuous renal replacement therapies), with single evaluation by a physician or other qualified health care professional |
|  | |  | procedure | | | UMLS:SNOMED:302497006 | Hemodialysis |
|  | |  | procedure | | | UMLS:CPT:1029674 | Dialysis Circuit Procedures |
|  | |  | procedure | | | UMLS:CPT:90935 | Hemodialysis procedure with single evaluation by a physician or other qualified health care professional |
|  | |  | procedure | | | UMLS:CPT:1006747 | Hemodialysis Access, Intervascular Cannulation for Extracorporeal Circulation, or Shunt Insertion Procedures on Arteries and Veins |
|  | |  | procedure | | | UMLS:CPT:1012740 | Dialysis Services and Procedures |
|  | |  | procedure | | | UMLS:CPT:1012752 | Hemodialysis Procedures |
|  | |  | procedure | | | UMLS:CPT:90947 | Dialysis procedure other than hemodialysis (eg, peritoneal dialysis, hemofiltration, or other continuous renal replacement therapies) requiring repeated evaluations by a physician or other qualified health care professional, with or without substantial revision of dialysis prescription |
|  | |  | procedure | | | UMLS:ICD9CM:39.95 | Hemodialysis |
|  | |  | diagnosis | | | UMLS:ICD10CM:Z99.2 | Dependence on renal dialysis |
|  | |  | procedure | | | UMLS:CPT:1012757 | Miscellaneous Dialysis Services and Procedures |
| date constraint | | | The terms in this group occurred between Jan 1, 2015 and May 1, 2023 | | | | |
| event relationship | | | Any instance of RRT occurred within 3 months and 7 days before any instance of Acute dialysis | | | | |
| Group 1B RRT before | | | | | | | |
| cannot have | |  | procedure | | | UMLS:ICD9CM:39.95 | Hemodialysis |
|  | | or | procedure | | | UMLS:CPT:90935 | Hemodialysis procedure with single evaluation by a physician or other qualified health care professional |
|  | | or | procedure | | | UMLS:CPT:1012752 | Hemodialysis Procedures |
|  | | or | procedure | | | UMLS:CPT:1006747 | Hemodialysis Access, Intervascular Cannulation for Extracorporeal Circulation, or Shunt Insertion Procedures on Arteries and Veins |
|  | | or | procedure | | | UMLS:CPT:90945 | Dialysis procedure other than hemodialysis (eg, peritoneal dialysis, hemofiltration, or other continuous renal replacement therapies), with single evaluation by a physician or other qualified health care professional |
|  | | or | procedure | | | UMLS:SNOMED:302497006 | Hemodialysis |
|  | | or | procedure | | | UMLS:CPT:1029674 | Dialysis Circuit Procedures |
|  | | or | procedure | | | UMLS:CPT:90947 | Dialysis procedure other than hemodialysis (eg, peritoneal dialysis, hemofiltration, or other continuous renal replacement therapies) requiring repeated evaluations by a physician or other qualified health care professional, with or without substantial revision of dialysis prescription |
|  | | or | procedure | | | UMLS:CPT:1012740 | Dialysis Services and Procedures |
|  | | or | diagnosis | | | UMLS:ICD10CM:Z99.2 | Dependence on renal dialysis |
|  | | or | procedure | | | UMLS:CPT:1012757 | Miscellaneous Dialysis Services and Procedures |
| Group 2A Acute dialysis | | | | | | | |
| must have | | any of | procedure | | | UMLS:CPT:90945 | Dialysis procedure other than hemodialysis (eg, peritoneal dialysis, hemofiltration, or other continuous renal replacement therapies), with single evaluation by a physician or other qualified health care professional |
|  | |  | procedure | | | UMLS:SNOMED:302497006 | Hemodialysis |
|  | |  | procedure | | | UMLS:CPT:1029674 | Dialysis Circuit Procedures |
|  | |  | procedure | | | UMLS:CPT:90935 | Hemodialysis procedure with single evaluation by a physician or other qualified health care professional |
|  | |  | procedure | | | UMLS:CPT:1006747 | Hemodialysis Access, Intervascular Cannulation for Extracorporeal Circulation, or Shunt Insertion Procedures on Arteries and Veins |
|  | |  | procedure | | | UMLS:CPT:1012740 | Dialysis Services and Procedures |
|  | |  | procedure | | | UMLS:CPT:1012752 | Hemodialysis Procedures |
|  | |  | procedure | | | UMLS:CPT:90947 | Dialysis procedure other than hemodialysis (eg, peritoneal dialysis, hemofiltration, or other continuous renal replacement therapies) requiring repeated evaluations by a physician or other qualified health care professional, with or without substantial revision of dialysis prescription |
|  | |  | procedure | | | UMLS:ICD9CM:39.95 | Hemodialysis |
|  | |  | diagnosis | | | UMLS:ICD10CM:Z99.2 | Dependence on renal dialysis |
|  | |  | procedure | | | UMLS:CPT:1012757 | Miscellaneous Dialysis Services and Procedures |
| date constraint | | | The terms in this group occurred at any time | | | | |
| event relationship | | | Any instance of GLP-1RAs occurred within 1 day before or up to 3 months after any instance of acute dialysis | | | | |
| Group 2B GLP-1RAs | | | | | | | |
| must have | |  | medication | | | NLM:ATC:A10BJ | Glucagon-like peptide-1 (GLP-1) analogues |
|  | | and | diagnosis | | | UMLS:ICD10CM:E11 | Type 2 diabetes mellitus |
| cannot have | |  | demographics | | | Deceased | Deceased |
|  | or | | | diagnosis | UMLS:ICD10CM:R99 | | Ill-defined and unknown cause of mortality |
|  | or | | | diagnosis | UMLS:ICD10CM:R69 | | Illness, unspecified |
|  | or | | | diagnosis | UMLS:ICD10CM:R99-R99 | | Ill-defined and unknown cause of mortality (R99) |

### Cohort of type 2 diabetes undergoing acute dialysis who did not receive GLP-1 RAs

| must have |  | demographics | Age | Age (between 18 and 90 years (most recent occurrence)) |
| --- | --- | --- | --- | --- |
| Group 1A Acute dialysis | | | | |
| must have | any of | procedure | UMLS:CPT:90945 | Dialysis procedure other than hemodialysis (eg, peritoneal dialysis, hemofiltration, or other continuous renal replacement therapies), with single evaluation by a physician or other qualified health care professional |
|  |  | procedure | UMLS:SNOMED:302497006 | Hemodialysis |
|  |  | procedure | UMLS:CPT:1029674 | Dialysis Circuit Procedures |
|  |  | procedure | UMLS:CPT:90935 | Hemodialysis procedure with single evaluation by a physician or other qualified health care professional |
|  |  | procedure | UMLS:CPT:1006747 | Hemodialysis Access, Intervascular Cannulation for Extracorporeal Circulation, or Shunt Insertion Procedures on Arteries and Veins |
|  |  | procedure | UMLS:CPT:1012740 | Dialysis Services and Procedures |
|  |  | procedure | UMLS:CPT:1012752 | Hemodialysis Procedures |
|  |  | procedure | UMLS:CPT:90947 | Dialysis procedure other than hemodialysis (eg, peritoneal dialysis, hemofiltration, or other continuous renal replacement therapies) requiring repeated evaluations by a physician or other qualified health care professional, with or without substantial revision of dialysis prescription |
|  |  | procedure | UMLS:ICD9CM:39.95 | Hemodialysis |
|  |  | diagnosis | UMLS:ICD10CM:Z99.2 | Dependence on renal dialysis |
|  |  | procedure | UMLS:CPT:1012757 | Miscellaneous Dialysis Services and Procedures |
| date constraint | | The terms in this group occurred between Jan 1, 2015 and May 1, 2023 | | |
| event relationship | | Any instance of RRT occurred within 3 months and 7 days before any instance of dialysis | | |
| Group 1B RRT before | | | | |
| cannot have |  | procedure | UMLS:ICD9CM:39.95 | Hemodialysis |
|  | or | procedure | UMLS:CPT:90935 | Hemodialysis procedure with single evaluation by a physician or other qualified health care professional |
|  | or | procedure | UMLS:CPT:1012752 | Hemodialysis Procedures |
|  | or | procedure | UMLS:CPT:1006747 | Hemodialysis Access, Intervascular Cannulation for Extracorporeal Circulation, or Shunt Insertion Procedures on Arteries and Veins |
|  | or | procedure | UMLS:CPT:90945 | Dialysis procedure other than hemodialysis (eg, peritoneal dialysis, hemofiltration, or other continuous renal replacement therapies), with single evaluation by a physician or other qualified health care professional |
|  | or | procedure | UMLS:SNOMED:302497006 | Hemodialysis |
|  | or | procedure | UMLS:CPT:1029674 | Dialysis Circuit Procedures |
|  | or | procedure | UMLS:CPT:90947 | Dialysis procedure other than hemodialysis (eg, peritoneal dialysis, hemofiltration, or other continuous renal replacement therapies) requiring repeated evaluations by a physician or other qualified health care professional, with or without substantial revision of dialysis prescription |
|  | or | procedure | UMLS:CPT:1012740 | Dialysis Services and Procedures |
|  | or | diagnosis | UMLS:ICD10CM:Z99.2 | Dependence on renal dialysis |
|  | or | procedure | UMLS:CPT:1012757 | Miscellaneous Dialysis Services and Procedures |
| Group 2A Acute dialysis | | | | |
| must have | any of | procedure | UMLS:CPT:90945 | Dialysis procedure other than hemodialysis (eg, peritoneal dialysis, hemofiltration, or other continuous renal replacement therapies), with single evaluation by a physician or other qualified health care professional |
|  |  | procedure | UMLS:SNOMED:302497006 | Hemodialysis |
|  |  | procedure | UMLS:CPT:1029674 | Dialysis Circuit Procedures |
|  |  | procedure | UMLS:CPT:90935 | Hemodialysis procedure with single evaluation by a physician or other qualified health care professional |
|  |  | procedure | UMLS:CPT:1006747 | Hemodialysis Access, Intervascular Cannulation for Extracorporeal Circulation, or Shunt Insertion Procedures on Arteries and Veins |
|  |  | procedure | UMLS:CPT:1012740 | Dialysis Services and Procedures |
|  |  | procedure | UMLS:CPT:1012752 | Hemodialysis Procedures |
|  |  | procedure | UMLS:CPT:90947 | Dialysis procedure other than hemodialysis (eg, peritoneal dialysis, hemofiltration, or other continuous renal replacement therapies) requiring repeated evaluations by a physician or other qualified health care professional, with or without substantial revision of dialysis prescription |
|  |  | procedure | UMLS:ICD9CM:39.95 | Hemodialysis |
|  |  | diagnosis | UMLS:ICD10CM:Z99.2 | Dependence on renal dialysis |
|  |  | procedure | UMLS:CPT:1012757 | Miscellaneous Dialysis Services and Procedures |
| date constraint | | The terms in this group occurred at any time | | |
| event relationship | | Any instance of receiving LAI occurred within 1 day before or up to 3 months after any instance of acute dialysis | | |
| Group 2B No GLP-1 RAs; Receiving LAI | | | | |
| must have |  | medication | NLM:ATC:A10AE | Insulins and analogues for injection, long-acting |
|  | and | diagnosis | UMLS:ICD10CM:E11 | Type 2 diabetes mellitus |
| cannot have |  | medication | NLM:ATC:A10BJ | Glucagon-like peptide-1 (GLP-1) analogues |
|  | or | demographics | Deceased | Deceased |
|  | or | diagnosis | UMLS:ICD10CM:R99 | Ill-defined and unknown cause of mortality |
|  | or | diagnosis | UMLS:ICD10CM:R69 | Illness, unspecified |
|  | or | diagnosis | UMLS:ICD10CM:R99-R99 | Ill-defined and unknown cause of mortality (R99) |

1. **Outcome definition**

| Mortality | | | | |
| --- | --- | --- | --- | --- |
|  | **Outcome definition** | | | |
|  | | Demographics | Deceased | Deceased |
|  | | Diagnosis | UMLS:ICD10CM:R99 | Ill-defined and unknown cause of mortality |
|  | | Diagnosis | UMLS:ICD10CM:R99-R99 | Ill-defined and unknown cause of mortality (R99) |
|  | | Diagnosis | UMLS:ICD10CM:R69 | Illness, unspecified |
|  | **Settings for the performed analyses** | | | |
|  | | Risk analysis | | including patients with outcome prior to the time window |
|  | | Kaplan - Meier survival analysis | | including patients with outcome prior to the time window |
|  | | Number of instances analysis | | including patients with outcome prior to the time window excluding patients with zero outcomes counts are grouped by date |
| 4p-MACE | | | | |
|  | **Outcome definition** | | | |
|  | | Demographics | Deceased | Deceased |
|  | | Diagnosis | UMLS:ICD10CM:R99 | Ill-defined and unknown cause of mortality |
|  | | Diagnosis | UMLS:ICD10CM:R99-R99 | Ill-defined and unknown cause of mortality (R99) |
|  | | Diagnosis | UMLS:ICD10CM:R69 | Illness, unspecified |
|  | | Diagnosis | UMLS:ICD10CM:I21 | Acute myocardial infarction |
|  | | Diagnosis | UMLS:ICD10CM:I63 | Cerebral infarction |
|  | | Diagnosis | UMLS:ICD10CM:I61 | Nontraumatic intracerebral hemorrhage |
|  | | Diagnosis | UMLS:ICD10CM:I46 | Cardiac arrest |
|  | | Procedure | UMLS:CPT:33533 | Coronary artery bypass, using arterial graft(s); single arterial graft |
|  | | Procedure | UMLS:CPT:1006217 | Coronary artery bypass, using arterial graft(s) |
|  | | Procedure | UMLS:CPT:33518 | Coronary artery bypass, using venous graft(s) and arterial graft(s); 2 venous grafts (List separately in addition to code for primary procedure) |
|  | | Procedure | UMLS:CPT:33517 | Coronary artery bypass, using venous graft(s) and arterial graft(s); single vein graft (List separately in addition to code for primary procedure) |
|  | | Procedure | UMLS:CPT:33519 | Coronary artery bypass, using venous graft(s) and arterial graft(s); 3 venous grafts (List separately in addition to code for primary procedure) |
|  | | Procedure | UMLS:CPT:1006208 | Coronary artery bypass, using venous graft(s) and arterial graft(s) |
|  | **Settings for the performed analyses** | | | |
|  | | Risk analysis | | excluding patients with outcome prior to the time window |
|  | | Kaplan - Meier survival analysis | | excluding patients with outcome prior to the time window |
|  | | Number of instances analysis | | excluding patients with outcome prior to the time window excluding patients with zero outcomes counts are grouped by date |
| MAKE | | | | |
|  | **Outcome definition** | | | |
|  | | Demographics | Deceased | Deceased |
|  | | Diagnosis | UMLS:ICD10CM:R99 | Ill-defined and unknown cause of mortality |
|  | | Diagnosis | UMLS:ICD10CM:R99-R99 | Ill-defined and unknown cause of mortality (R99) |
|  | | Diagnosis | UMLS:ICD10CM:R69 | Illness, unspecified |
|  | | Procedure | UMLS:CPT:90947 | Dialysis procedure other than hemodialysis (eg, peritoneal dialysis, hemofiltration, or other continuous renal replacement therapies) requiring repeated evaluations by a physician or other qualified health care professional, with or without substantial revision of dialysis prescription |
|  | | Procedure | UMLS:CPT:90935 | Hemodialysis procedure with single evaluation by a physician or other qualified health care professional |
|  | | Procedure | UMLS:CPT:90945 | Dialysis procedure other than hemodialysis (eg, peritoneal dialysis, hemofiltration, or other continuous renal replacement therapies), with single evaluation by a physician or other qualified health care professional |
|  | | Procedure | UMLS:CPT:1012752 | Hemodialysis Procedures |
|  | | Procedure | UMLS:CPT:1012740 | Dialysis Services and Procedures |
|  | | Procedure | UMLS:CPT:1029674 | Dialysis Circuit Procedures |
|  | | Procedure | UMLS:ICD9CM:39.95 | Hemodialysis |
|  | | Procedure | UMLS:SNOMED:302497006 | Hemodialysis |
|  | **Settings for the performed analyses** | | | |
|  | | Kaplan - Meier survival analysis | | including patients with outcome prior to the time window |
|  | | Number of instances analysis | | including patients with outcome prior to the time window excluding patients with zero outcomes counts are grouped by date |
|  | | Risk analysis | | including patients with outcome prior to the time window |

| Gastroparesis | | | |
| --- | --- | --- | --- |
| Outcome definition | | | |
|  | Diagnosis | UMLS:ICD10CM:K31.84 | Gastroparesis |
|  | Diagnosis | UMLS:ICD10CM:E11.40 | Type 2 diabetes mellitus with diabetic neuropathy, unspecified |
| Settings for the performed analyses | | | |
|  | Risk analysis | | excluding patients with outcome prior to the time window |
|  | Kaplan - Meier survival analysis | | excluding patients with outcome prior to the time window |
| Tachycardia | | | |
| Outcome definition | | | |
|  | Diagnosis | UMLS:ICD10CM:R00.0 | Tachycardia, unspecified |
|  | Diagnosis | UMLS:ICD10CM:I47 | Paroxysmal tachycardia |
|  | Diagnosis | UMLS:ICD10CM:I47.29 | Other ventricular tachycardia |
|  | Diagnosis | UMLS:ICD10CM:I47.20 | Ventricular tachycardia, unspecified |
|  | Diagnosis | UMLS:ICD10CM:I47.10 | Supraventricular tachycardia, unspecified |
|  | Diagnosis | UMLS:ICD10CM:I47.2 | Ventricular tachycardia |
|  | Diagnosis | UMLS:ICD10CM:I47.1 | Supraventricular tachycardia |
| Settings for the performed analyses | | | |
|  | Risk analysis | | excluding patients with outcome prior to the time window |
|  | Kaplan - Meier survival analysis | | excluding patients with outcome prior to the time window |

| Hernia | | | | | | | | | | |
| --- | --- | --- | --- | --- | --- | --- | --- | --- | --- | --- |
|  | Outcome definition | | | | | | | | | |
|  | | | | Diagnosis | UMLS:ICD10CM:K40-K46 | | | | | Hernia |
|  | Settings for the performed analyses | | | | | | | | | |
|  | | | | Risk analysis | | | | | | excluding patients with outcome prior to the time window |
|  | | | | Kaplan– - Meier survival analysis | | | | | | excluding patients with outcome prior to the time window |
|  | | | | Number of instances analysis | | | | | | excluding patients with outcome prior to the time window excluding patients with zero outcomes counts are grouped by date |
| Traumatic brain injury | | | | | | | | | | |
|  | Outcome definition | | | | | | | | | |
|  | | | | Diagnosis | UMLS:ICD10CM:S06.5 | | | | | Traumatic subdural hemorrhage |
|  | | | | Diagnosis | UMLS:ICD10CM:S06.6 | | | | | Traumatic subarachnoid hemorrhage |
|  | | | | Diagnosis | UMLS:ICD10CM:S06.2 | | | | | Diffuse traumatic brain injury |
|  | | | | Diagnosis | UMLS:ICD10CM:S06.3 | | | | | Focal traumatic brain injury |
|  | Settings for the performed analyses | | | | | | | | | |
|  | | | | Risk analysis | | | | | | excluding patients with outcome prior to the time window |
|  | | | | Kaplan - Meier survival analysis | | | | | | excluding patients with outcome prior to the time window |
| Melanoma | | | | | | | | | | |
|  | Outcome definition | | | | | | | | | |
|  | | | | Diagnosis | UMLS:ICD10CM:C43 | | | | | Malignant melanoma of skin |
|  | Settings for the performed analyses | | | | | | | | | |
|  | | | | Risk analysis | | | | | | excluding patients with outcome prior to the time window |
|  | | | | Kaplan - Meier survival analysis | | | | | | excluding patients with outcome prior to the time window |
| Pneumonia | | | | | | | | | | |
| Outcome definition | | | | | | | | | | |
|  | | Diagnosis | | | | UMLS:ICD10CM:J18 | | | Pneumonia, unspecified organism | |
|  | | Diagnosis | | | | UMLS:ICD10CM:J18.9 | | | Pneumonia, unspecified organism | |
|  | | Diagnosis | | | | UMLS:ICD10CM:J12.82 | | | Pneumonia due to coronavirus disease 2019 | |
|  | | Diagnosis | | | | UMLS:ICD10CM:J15.2 | | | Pneumonia due to staphylococcus | |
|  | | Diagnosis | | | | UMLS:ICD10CM:J15.21 | | | Pneumonia due to staphylococcus aureus | |
|  | | Diagnosis | | | | UMLS:ICD10CM:J16 | | | Pneumonia due to other infectious organisms, not elsewhere classified | |
|  | | Diagnosis | | | | UMLS:ICD10CM:J15.6 | | | Pneumonia due to other Gram-negative bacteria | |
|  | | Diagnosis | | | | UMLS:ICD10CM:J13 | | | Pneumonia due to Streptococcus pneumoniae | |
|  | | Diagnosis | | | | UMLS:ICD10CM:J15.7 | | | Pneumonia due to Mycoplasma pneumoniae | |
|  | | Diagnosis | | | | UMLS:ICD10CM:J15.1 | | | Pneumonia due to Pseudomonas | |
|  | | Diagnosis | | | | UMLS:ICD10CM:J15.8 | | | Pneumonia due to other specified bacteria | |
|  | | Diagnosis | | | | UMLS:ICD10CM:J15.212 | | | Pneumonia due to Methicillin resistant Staphylococcus aureus | |
|  | | Diagnosis | | | | UMLS:ICD10CM:J15.211 | | | Pneumonia due to Methicillin susceptible Staphylococcus aureus | |
|  | | Diagnosis | | | | UMLS:ICD10CM:J15.0 | | | Pneumonia due to Klebsiella pneumoniae | |
|  | | Diagnosis | | | | UMLS:ICD10CM:J15.4 | | | Pneumonia due to other streptococci | |
|  | | Diagnosis | | | | UMLS:ICD10CM:J14 | | | Pneumonia due to Hemophilus influenzae | |
| Settings for the performed analyses | | | | | | | | | | |
|  | | Risk analysis | | | | | | | excluding patients with outcome prior to the time window | |
|  | | Kaplan - Meier survival analysis | | | | | | | excluding patients with outcome prior to the time window | |
| GI bleeding | | | | | | | | | | |
| Outcome definition | | | | | | | | | | |
|  | | | Diagnosis | | | | UMLS:ICD10CM:K92 | Other diseases of digestive system | | |
|  | | | Diagnosis | | | | UMLS:ICD10CM:R58 | Hemorrhage, not elsewhere classified | | |
|  | | | Diagnosis | | | | UMLS:ICD10CM:K62.5 | Hemorrhage of anus and rectum | | |
| Settings for the performed analyses | | | | | | | | | | |
|  | | | Risk analysis | | | | | excluding patients with outcome prior to the time window | | |
|  | | | Kaplan - Meier survival analysis | | | | | excluding patients with outcome prior to the time window | | |

1. **Supplementary table**

**Suppl. Table 1. The most common causes of AKI**

|  | **All patients**  **(n=34,435)** | **GLP-1RAs group**  **(n=1,685)** | **LAI group**  **(n=32,750)** | ***P-value*** |
| --- | --- | --- | --- | --- |
| Ischemic heart disease | 5,915 (17.2%) | 246 (14.6%) | 5,669 (17.3%) | < 0.001 |
| Heart failure | 4,688 (13.6%) | 188 (11.1%) | 4,500 (13.7%) | 0.007 |
| Cardiogenic shock | 426 (1.2%) | 22 (1.3%) | 404 (1.2%) | 0.845 |
| Hypertension crisis | 592 (1.7%) | 15 (0.9%) | 577 (1.8%) | 0.006 |
| Hypovolemic shock | 205 (0.6%) | 10 (0.6%) | 195 (0.6%) | 0.964 |
| Sepsis | 2,254 (6.5%) | 47 (2.8%) | 2,207 (6.7%) | < 0.001 |
| Septic shock | 1,043 (3.0%) | 18 (1.1%) | 1,025 (3.1%) | < 0.001 |
| Urinary tract infection | 1,694 (4.9%) | 51 (3.0%) | 1,643 (5.0%) | < 0.001 |
| Obstructive uropathy | 519 (1.5%) | 18 (1.1%) | 501 (1.5%) | 0.116 |
| Acute tubular necrosis | 1,781 (5.2%) | 40 (2.4%) | 1,741 (5.3%) | < 0.001 |
| Nephrotic syndrome | 110 (0.3%) | 10 (0.6%) | 100 (0.3%) | 0.045 |
| eGFR <15 [mL/min/1.73m^2^] | 8,618 (25.0%) | 247 (14.7%) | 8,371 (25.6%) | < 0.001 |

**Abbreviation:** AKI, acute kidney injury; eGFR, estimated glomerular filtration rate; GLP-1RAs, glucagon-like peptide receptor agonists; LAI, long‐acting insulins.

**Suppl. Table 2. Follow-up period of our analysis for overall cohort.**

| **Outcomes** | **Follow-up period (year)** | | |  |
| --- | --- | --- | --- | --- |
| **Mortality** | **All patients (n=3,364)** | **GLP-1RAs group (n=1,682)** | **LAI group (n=1,682)** |  |
| Mean | 2.0 | 2.1 | 2.0 |  |
| Median [Q1 - Q3] | 1.4 [0.8 - 2.1] | 1.4 [0.8 - 2.1] | 1.4 [0.8 - 2.1] |  |
| 90th percentile | 2.6 | 2.6 | 2.6 |  |
|  |  |  |  |  |
| **4P-MACE** | **All patients (n=2,482)** | **GLP-1RAs group (n=1,253)** | **LAI group (n=1,229)** |  |
| Mean | 1.7 | 1.8 | 1.7 |  |
| Median [Q1 - Q3] | 1.3 [0.7 - 2.0] | 1.3 [0.7 - 2.0] | 1.3 [0.7 - 2.0] |  |
| 90th percentile | 2.6 | 2.6 | 2.5 |  |
|  |  |  |  |  |
| **MAKE** | **All patients (n=3,364)** | **GLP-1RAs group (n=1,682)** | **LAI group (n=1,682)** |  |
| Mean | 1.8 | 1.9 | 1.8 |  |
| Median [Q1 - Q3] | 1.3 [0.7 - 2.0] | 1.3 [0.7 - 2.1] | 1.3 [0.7 - 2.0] |  |
| 90th percentile | 2.6 | 2.6 | 2.6 |  |

**Abbreviations:** 4P-MACE, four-point major adverse cardiac events; MAKE, major adverse kidney events.

**Suppl. Table 3. Risk of mortality in type 2 diabetes patients undergoing acute dialysis**: **comparison between GLP-1RAs users and LAI after propensity score matching.** The results presented in the table below are from an analysis of a cohort of patients after propensity score matching.

| **1 Mortality** | | | | | | | | | | | | |
| --- | --- | --- | --- | --- | --- | --- | --- | --- | --- | --- | --- | --- |
|  | | **Risk analysis** | | | | | | | | | | |
|  |  | | | Cohort | | | Patients in cohort | Patients with outcome | Risk | | | |
|  | | |  | 1 | | Type2 DM-acute dialysis-GLP-1RAs | 1,682 | 110 | 0.065 | | | |
|  | | |  | 2 | | Type2 DM-acute dialysis-LAI | 1,682 | 185 | 0.110 | | | |
|  | | | | | | | | | | | | |
|  | | |  |  | | |  | 95% CI | z | p |  |  |
|  | | |  | **Risk Difference** | | | -0.045 | (-0.064, -0.026) | -4.572 | 0.000 |  |  |
|  | | |  | **Risk Ratio** | | | 0.595 | (0.474, 0.745) | N/A | N/A |  |  |
|  | | |  | **Odds Ratio** | | | 0.566 | (0.443, 0.724) | N/A | N/A |  |  |
|  | | | | | | | | | | | | |
|  | |  | | | 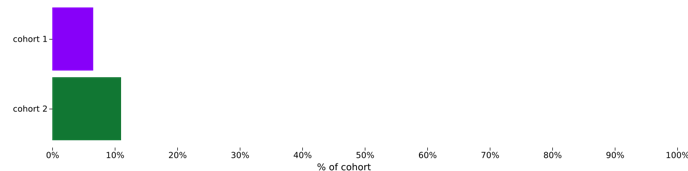 | | | | | | | |
|  | | **Kaplan - Meier survival analysis** | | | | | | | | | | |
|  | | |  | Cohort | | | Patients in cohort | Patients with outcome | Median survival (days) | Survival probability at end of time window | | |
|  | | |  | 1 | | Type2 DM-acute dialysis-GLP-1RAs | 1,682 | 110 | -- | 86.57% | | |
|  | | |  | 2 | | Type2 DM-acute dialysis-LAI | 1,682 | 185 | -- | 81.60% | | |
|  | | | | | | | | | | | | |
|  | | |  |  | | | χ^2^ | df | p |  |  |  |
|  | | |  | **Log-Rank Test** | | | 14.600 | 1 | 0.000 |  |  |  |
|  | | | | | | | | | | | | |
|  | | |  |  | | | Hazard Ratio | 95% CI | χ^2^ | df | p | |
|  | | |  | **Hazard Ratio and Proportionality** | | | 0.633 | (0.500, 0.802) | 6.133 | 1 | 0.013 | |
|  | |  | | | 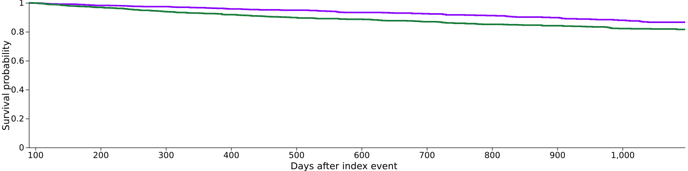 | | | | | | | |
|  | | **Number of instances** | | | | | | | | | | |
|  | | |  | Cohort | | | Patients in cohort | Patients with outcome | Mean | Standard Deviation | Median | |
|  | | |  | 1 | | Type2 DM-acute dialysis-GLP-1RAs | 1,682 | 110 | 1.291 | 1.026 | 1 | |
|  | | |  | 2 | | Type2 DM-acute dialysis-LAI | 1,682 | 185 | 1.378 | 2.807 | 1 | |
|  | | | | | | | | | | | | |
|  | | |  |  | | | t | df | p |  |  |  |
|  | | |  | **Test Statistics** | | | -0.314 | 293 | 0.753 |  |  |  |
|  | |  | | | 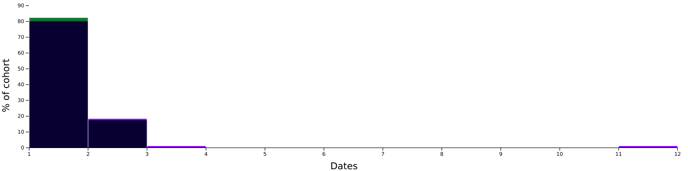 | | | | | | | |
|  | |  | | | 0 data points for Cohort 1 and 1 data points for Cohort 2 were omitted for display purposes. | | | | | | | |

**Suppl. Table 4. Risk of MACE in type 2 diabetes patients undergoing acute dialysis**: **comparison between GLP-1RAs users and LAI users after propensity score matching.**The results presented in the table below are from an analysis of a cohort of patients after propensity score matching. The baseline cohort included patients with a history of MACE, who were excluded from the analysis

| **2 4p-MACE** | | | | | | | | | | | |
| --- | --- | --- | --- | --- | --- | --- | --- | --- | --- | --- | --- |
| **Risk analysis excluding patients with outcome prior to the time window** | | | | | | | | | | | |
|  | | | Cohort | | | Patients in cohort | Patients with outcome | Risk | | | |
|  |  | | | 1 | Type2 DM-acute dialysis-GLP-1RAs | 1,253 | 115 | 0.092 | | | |
|  |  | | | 2 | Type2 DM-acute dialysis-LAI | 1,229 | 182 | 0.148 | | | |
|  | | | | | | | | | | | |
|  |  | | |  | |  | 95% CI | z | p |  |  |
|  |  | | | **Risk Difference** | | -0.056 | (-0.082, -0.031) | -4.321 | 0.000 |  |  |
|  |  | | | **Risk Ratio** | | 0.620 | (0.497, 0.772) | N/A | N/A |  |  |
|  |  | | | **Odds Ratio** | | 0.581 | (0.454, 0.745) | N/A | N/A |  |  |
|  | | | | | | | | | | | |
|  | | 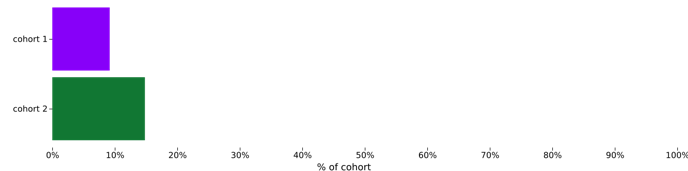 | | | | | | | | | |
|  | | 429 patients in Cohort 1 and 453 patients in Cohort 2 were excluded from results because they had the outcome prior to the time window. | | | | | | | | | |
| **Kaplan - Meier survival analysis excluding patients with outcome prior to the time window** | | | | | | | | | | | |
|  |  | | | Cohort | | Patients in cohort | Patients with outcome | Median survival (days) | Survival probability at end of time window | | |
|  |  | | | 1 | Type2 DM-acute dialysis-GLP-1RAs | 1,253 | 115 | -- | 82.18% | | |
|  |  | | | 2 | Type2 DM-acute dialysis-LAI | 1,229 | 182 | -- | 75.11% | | |
|  | | | | | | | | | | | |
|  |  | | |  | | χ^2^ | df | p |  |  |  |
|  |  | | | **Log-Rank Test** | | 13.698 | 1 | 0.000 |  |  |  |
|  | | | | | | | | | | | |
|  |  | | |  | | Hazard Ratio | 95% CI | χ^2^ | df | p | |
|  |  | | | **Hazard Ratio and Proportionality** | | 0.645 | (0.511, 0.815) | 1.680 | 1 | 0.195 | |
|  | | | | | | | | | | | |
|  | | 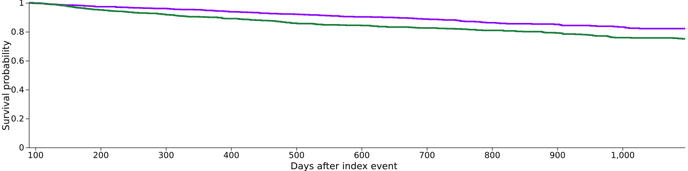 | | | | | | | | | |
|  | | 429 patients in Cohort 1 and 453 patients in Cohort 2 were excluded from results because they had the outcome prior to the time window. | | | | | | | | | |
| **Number of instances excluding patients with outcome prior to the time window** | | | | | | | | | | | |
|  |  | | | Cohort | | Patients in cohort | Patients with outcome | Mean | Standard Deviation | Median | |
|  |  | | | 1 | Type2 DM-acute dialysis-GLP-1RAs | 1,253 | 115 | 2.017 | 2.899 | 1 | |
|  |  | | | 2 | Type2 DM-acute dialysis-LAI | 1,229 | 182 | 2.593 | 4.744 | 1 | |
|  | | | | | | | | | | | |
|  |  | | |  | | t | df | p |  |  |  |
|  |  | | | **Test Statistics** | | -1.171 | 295 | 0.243 |  |  |  |
|  | | | | | | | | | | | |
|  | | 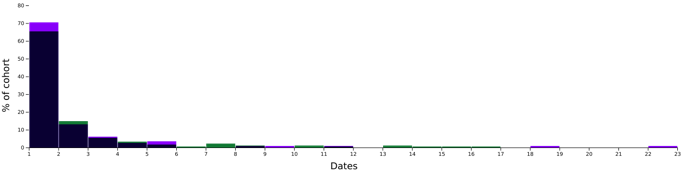 | | | | | | | | | |
|  | | 0 data points for Cohort 1 and 2 data points for Cohort 2 were omitted for display purposes. 429 patients in Cohort 1 and 453 patients in Cohort 2 were excluded from results because they had the outcome prior to the time window. | | | | | | | | | |

**Suppl. Table 5. Risk of MAKE in type 2 diabetes patients undergoing acute dialysis**: **comparison between GLP-1RAs users and LAI users after propensity score matching.** The results presented in the table below are from an analysis of a cohort of patients after propensity score matching. The baseline cohort included patients with a history of MAKE, who were excluded from the analysis

| **3 MAKE** | | | | | | | | | | | | |
| --- | --- | --- | --- | --- | --- | --- | --- | --- | --- | --- | --- | --- |
|  | | **Risk analysis** | | | | | | | | | | |
|  |  | | | Cohort | | | Patients in cohort | Patients with outcome | Risk | | | |
|  | | |  | 1 | | Type2 DM-acute dialysis-GLP-1RAs | 1,682 | 270 | 0.161 | | | |
|  | | |  | 2 | | Type2 DM-acute dialysis-LAI | 1,682 | 364 | 0.216 | | | |
|  | | | | | | | | | | | | |
|  | | |  |  | | |  | 95% CI | z | p |  |  |
|  | | |  | **Risk Difference** | | | -0.056 | (-0.082, -0.030) | -4.144 | 0.000 |  |  |
|  | | |  | **Risk Ratio** | | | 0.742 | (0.643, 0.855) | N/A | N/A |  |  |
|  | | |  | **Odds Ratio** | | | 0.692 | (0.582, 0.824) | N/A | N/A |  |  |
|  | | | | | | | | | | | | |
|  | |  | | | 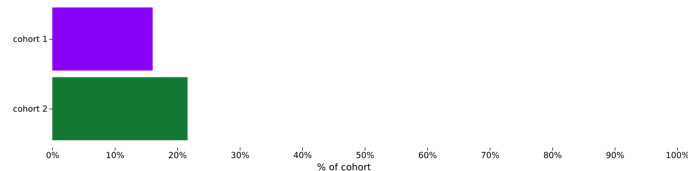 | | | | | | | |
|  | | **Kaplan - Meier survival analysis** | | | | | | | | | | |
|  | | |  | Cohort | | | Patients in cohort | Patients with outcome | Median survival (days) | Survival probability at end of time window | | |
|  | | |  | 1 | | Type2 DM-acute dialysis-GLP-1RAs | 1,682 | 270 | -- | 70.71% | | |
|  | | |  | 2 | | Type2 DM-acute dialysis-LAI | 1,682 | 364 | -- | 65.42% | | |
|  | | | | | | | | | | | | |
|  | | |  |  | | | χ^2^ | df | p |  |  |  |
|  | | |  | **Log-Rank Test** | | | 13.327 | 1 | 0.000 |  |  |  |
|  | | | | | | | | | | | | |
|  | | |  |  | | | Hazard Ratio | 95% CI | χ^2^ | df | p | |
|  | | |  | **Hazard Ratio and Proportionality** | | | 0.746 | (0.638, 0.874) | 6.040 | 1 | 0.014 | |
|  | | | | | | | | | | | | |
|  | |  | | | 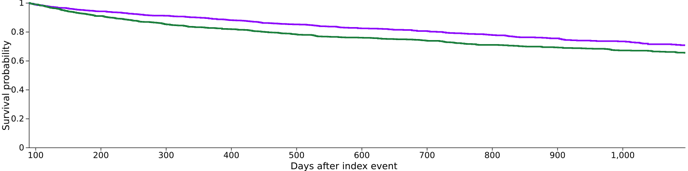 | | | | | | | |
|  | | **Number of instances** | | | | | | | | | | |
|  | | |  | Cohort | | | Patients in cohort | Patients with outcome | Mean | Standard Deviation | Median | |
|  | | |  | 1 | | Type2 DM-acute dialysis-GLP-1RAs | 1,682 | 270 | 2.867 | 5.456 | 1 | |
|  | | |  | 2 | | Type2 DM-acute dialysis-LAI | 1,682 | 364 | 2.445 | 4.167 | 1 | |
|  | | | | | | | | | | | | |
|  | | |  |  | | | t | df | p |  |  |  |
|  | | |  | **Test Statistics** | | | 1.103 | 632 | 0.270 |  |  |  |
|  | | | | | | | | | | | | |
|  | |  | | | 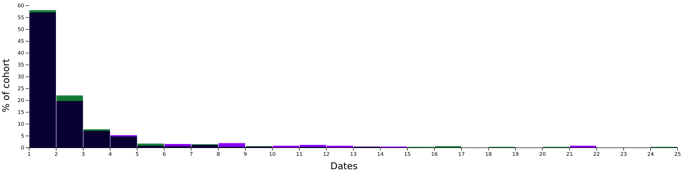 | | | | | | | |
|  | |  | | | 4 data points for Cohort 1 and 4 data points for Cohort 2 were omitted for display purposes. | | | | | | | |

**Suppl. Table 6. Risk of all-cause mortality, MACE, and MAKE between exenatide users and LAI in type 2 diabetes patients undergoing acute dialysis**

| **Exenatide users group versus LAI users group** |  | **aHR (95%CI)** | **P-value** |
| --- | --- | --- | --- |
| **Primary outcome** |  |  |  |
| All-cause mortality |  | 0.45 (0.15-1.38) | 0.154 |
| **Secondary outcome** |  |  |  |
| 4P-MACE |  | 0.48 (0.16-1.48) | 0.193 |
| MAKE |  | 0.47 (0.13-1.66) | 0.230 |

**Abbreviations:** aHR, adjusted hazard ratios; CI, confidence interval; LAI, long‐acting insulins; 4P-MACE, four-point major adverse cardiac events; MAKE, major adverse kidney events.

**Suppl. Table 7. Positive exposure control for all-cause mortality, MACE, and MAKE between ACEI/ARB user and ACEI/ARB non-user in a new-user design.**

| **Outcome** | **Patients with outcome** | | **aHR (95%CI)** |  |  |
| --- | --- | --- | --- | --- | --- |
|  | **ACEI/ARB users group** | **ACEI/ARB non-users** |  | |  |
| **Primary outcome** |  |  |  | |  |
| Mortality | 10.3% (2,273/ 22,051) | 12.4% (2,733/ 22,051) | 0.82 (0.78- 0.87) | |  |
| **Secondary outcome** |  |  |  | |  |
| 4P-MACE | 13.2% (2,140/ 16,172) | 14.0% (2,326/ 16,565) | 0.94 (0.88- 0.99) | |  |
| MAKE | 22.6% (4,981/ 22,051) | 20.6% (4,553/ 22,051) | 1.12 (1.08-1.17) | |  |

**Abbreviations:** aHR, adjusted hazard ratios; ACEI, angiotensin converting enzyme inhibitor; ARB, angiotensin Ⅱ receptor blocker; CI, confidence interval; 4P-MACE, four-point major adverse cardiac events; MAKE, major adverse kidney events.

**Suppl. Table 8. Landmark Analysis, Cumulative probability for the risk of all-cause mortality, 4P-MACE, and MAKE within different selective duration of GLP-1RAs.**

| **Duration of GLP‐1RAs usage within 90 days** |  | **aHR (95%CI)** | **P-value** |
| --- | --- | --- | --- |
| **14-Day Duration** |  |  |  |
| All-cause mortality |  | 0.58 (0.45-0.75) | <0.001 |
| 4P-MACE |  | 0.73 (0.56-0.95) | 0.018 |
| MAKE |  | 0.71 (0.59-0.85) | <0.001 |
| **30-Day Duration** |  |  |  |
| All-cause mortality |  | 0.63 (0.49-0.81) | <0.001 |
| 4P-MACE |  | 0.64 (0.50-0.83) | <0.001 |
| MAKE |  | 0.80 (0.67-0.94) | 0.008 |
| **60-Day Duration** |  |  |  |
| All-cause mortality |  | 0.68 (0.54-0.86) | 0.002 |
| 4P-MACE |  | 0.71 (0.56-0.91) | 0.006 |
| MAKE |  | 0.78 (0.66-0.91) | 0.002 |
| **90-Day Duration** |  |  |  |
| All-cause mortality |  | 0.63 (0.50-0.80) | <0.001 |
| 4P-MACE |  | 0.65 (0.51-0.81) | <0.001 |
| MAKE |  | 0.75 (0.64-0.87) | <0.001 |

**Abbreviations:** aHR, adjusted hazard ratio; CI, confidence interval; GLP-1RAs, glucagon-like peptide receptor agonists; 4P-MACE, 4-point major adverse cardiovascular events; MAKE, major adverse kidney events.

**Suppl. Table 9. Changes of HbA1c, body weight, low-density lipoprotein (LDL), and systolic blood pressure (SBP) of patients treated with GLP-1RAs and LAI at baseline, day-30-60, and day-60-90**

| **Duration within 90 days** | **GLP-1RAs group**  **(Mean± SD)** | **LAI group**  **(Mean± SD)** | **P-value** |
| --- | --- | --- | --- |
| **HbA1c** |  |  |  |
| Baseline | 7.96 ± 2.08 | 7.88 ± 2.07 | 0.177 |
| 30-60 Day Duration | 7.57 ± 1.60 | 7.44 ± 1.64 | 0.498 |
| 60-90 Day Duration | 7.63 ± 1.82 | 7.25 ± 1.84 | **0.049** |
| **Body weight** |  |  |  |
| Baseline | 208 ± 57.90 | 204 ± 61.2 | 0.083 |
| 30-60 Day Duration | 208.34 ± 55.96 | 195.33 ± 54.50 | **<0.001** |
| 60-90 Day Duration | 207.55 ± 53.06 | 200.21 ± 53.54 | **0.043** |
| **LDL** |  |  |  |
| Baseline | 83 ± 39.30 | 82.6 ± 41.1 | 0.994 |
| 30-60 Day Duration | 79.34 ± 37.09 | 80.61 ± 38.39 | 0.800 |
| 60-90 Day Duration | 84.47 ± 46.15 | 81.27 ± 39.79 | 0.573 |
| **SBP** |  |  |  |
| Baseline | 132 ± 23.7 | 129 ± 28.6 | 0.443 |
| 30-60 Day Duration | 131.96 ± 20.65 | 131.03 ± 22.45 | 0.526 |
| 60-90 Day Duration | 134.25 ± 21.48 | 131.37 ± 23.34 | 0.075 |

**Abbreviations:** GLP-1RAs, glucagon-like peptide receptor agonists; LAI, long‐acting insulins; LDL, low-density lipoprotein; SBP, systolic blood pressure; SD, standard deviation.

**Suppl. Table 10.** **Incidence of outcomes among GLP-1RAs group compared to LAI control group after adjustment for diabetic retinopathy and neuropathy, as proxies for disease severity**

|  |  | **aHR (95%CI)** | **P-value** |
| --- | --- | --- | --- |
| **Primary outcome** |  |  |  |
| All-cause mortality |  | 0.56 (0.44-0.71) | <0.001 |
| **Secondary outcome** |  |  |  |
| 4P-MACE |  | 0.69 (0.54-0.87) | 0.001 |
| MAKE |  | 0.76 (0.65-0.88) | <0.001 |

**Abbreviations:** aHR, adjusted hazard ratio; GLP-1RAs, glucagon-like peptide-1 receptor agonists; LAI, long-acting insulin; 4P-MACE, four-point major adverse cardiovascular event; MAKE; major adverse kidney event.

**Suppl. Table 11. Risk of all-cause mortality, MACE, and MAKE between GLP-1RAs users and patients using dipeptidyl peptidase-4 inhibitor (DPP4i), thiazolidinedione (TZD) or sulfonylureas in type 2 diabetes patients undergoing acute dialysis**

|  |  | **aHR (95%CI)** | **P-value** |
| --- | --- | --- | --- |
| **GLP-1RAs versus DPP4i/ SU/ TZD** |  |  |  |
| All-cause mortality |  | 0.71 (0.55 -0.90) | 0.005 |
| 4P-MACE |  | 0.76 (0.60-0.97) | 0.028 |
| MAKE |  | 0.84 (0.72-0.98) | 0.031 |

**Abbreviations:** aHR, adjusted hazard ratio; CI, confidence interval; DPP4i, dipeptidyl peptidase-4 inhibitor; L; GLP-1RAs, glucagon-like peptide receptor agonists; LAI, long‐acting insulins; 4P-MACE, four-point major adverse cardiac events; MAKE, major adverse kidney events; TZD, thiazolidinedione.

**Suppl. Table 12. Risk of hypoglycemia in type 2 diabetes patients undergoing acute dialysis**: **between GLP-1RAs users and LAI users after propensity score matching.**

| **Outcome** | **Patients with outcome** | | **aHR (95%CI)** | **P-value** |
| --- | --- | --- | --- | --- |
|  | **GLP-1RAs group** | **LAI group** |  |  |
| **Hypoglycemia** |  |  |  |  |
| Patients with outcome | 6.1% (73/ 1,202) | 4.8% (58/ 1,218) | 1.33 (0.94-1.88) | 0.102 |

**Abbreviations:** aHR, adjusted hazard ratio; CI, confidence interval; GLP-1RAs, glucagon-like peptide receptor agonists; LAI, long‐acting insulins.

1. **Supplementary figures**

**Suppl. Figure 1. Positive Outcomes Control, Negative Outcomes Control, and Positive Exposure Control**


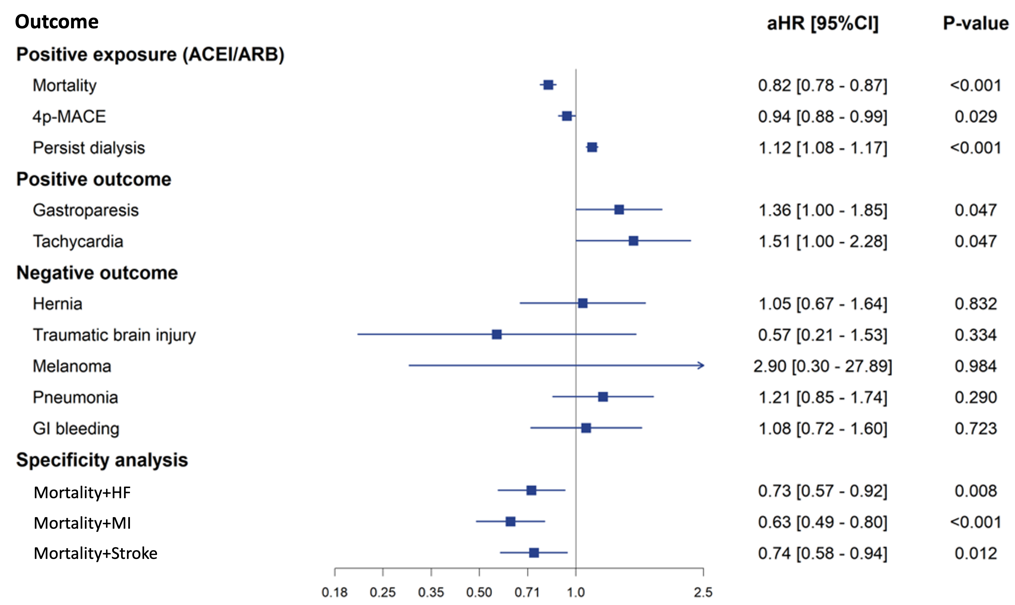


**Abbreviations:** ACEI, angiotensin-converting enzyme inhibitors; aHR, adjusted hazard ratio; ARB, angiotensin-receptor blockers; CI, confidence interval; HF, heart failure; MI, myocardial infarction; 4P-MACE, 4-point major adverse cardiovascular events.

**Suppl. Figure 2. Comparison of Effect of Glycemic control between GLP-1RAs users and LAI group at baseline, day-30-60, and day-60-90**

**
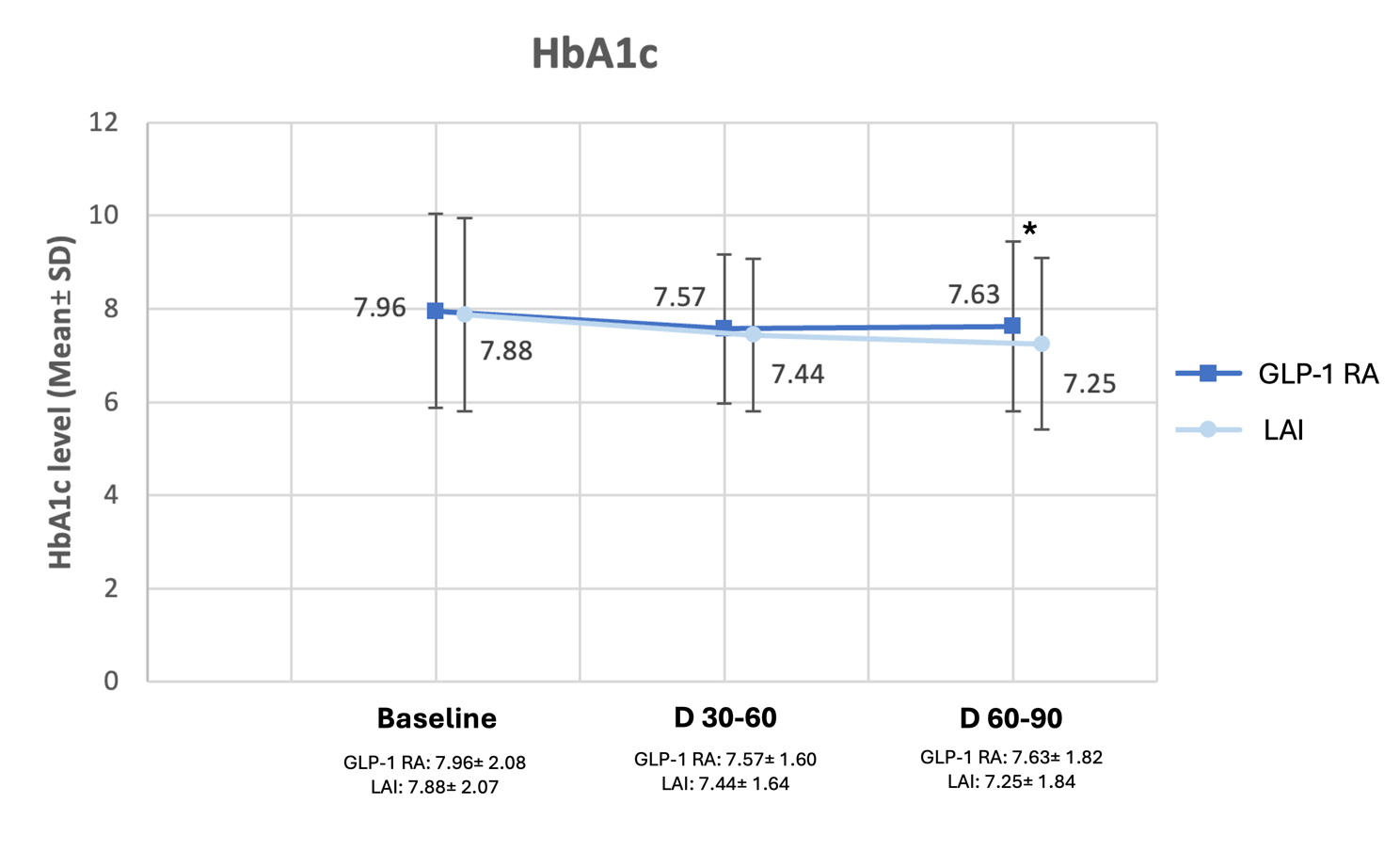
**

**Abbreviations:** GLP-1RAs, glucagon-like peptide receptor agonists; LAI, long‐acting insulins; SD, standard deviation. The p-values are indicated as follows: *p<0.05, **p<0.01. Graphical data are shown as mean ± SD.

**Suppl. Figure 3: Changes of body weight of patients treated with GLP-1RAs and LAI at baseline, day-30-60, and day-60-90

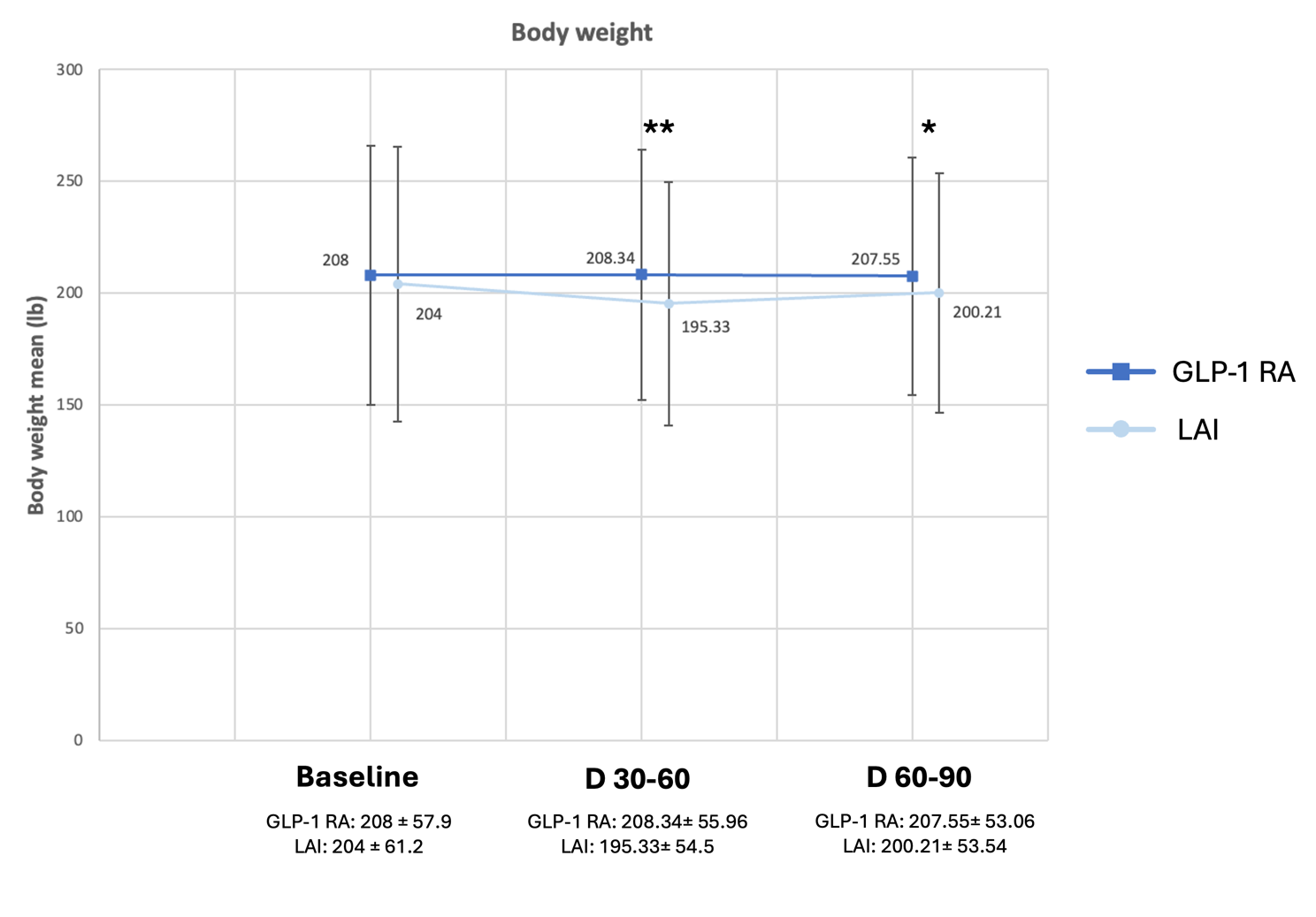
**

**Abbreviations:** GLP-1RAs, glucagon-like peptide receptor agonists; LAI, long‐acting insulin. The p-values are indicated as follows: *p<0.05, **p<0.01. Graphical data are shown as mean ± SD.

**Suppl. Figure 4. Changes of low-density lipoprotein (LDL) of patients treated with GLP-1RAs and LAI at baseline, day-30-60, and day-60-90**

**
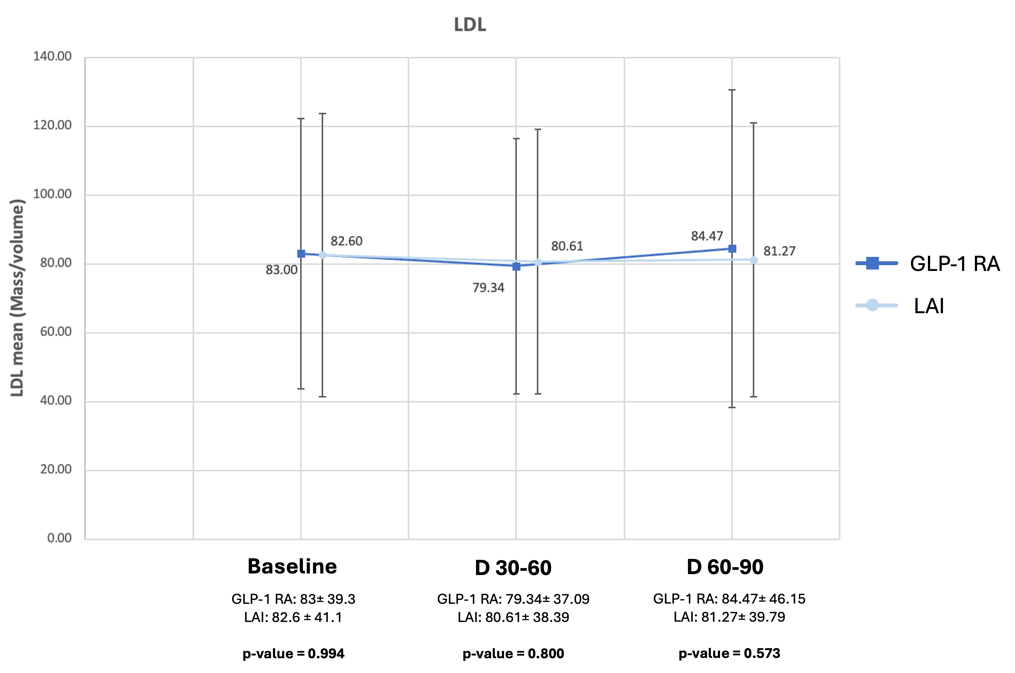
**

**Abbreviations:** GLP-1RAs, glucagon-like peptide receptor agonists; LAI, long‐acting insulin; LDL, low-density lipoprotein. The p-values are indicated as follows: *p<0.05, **p<0.01. Graphical data are shown as mean ± SD.

**Suppl. Figure 5. Changes of systolic blood pressure (SBP) of patients treated with GLP-1RAs and LAI at baseline, day-30-60, and day-60-90**

**
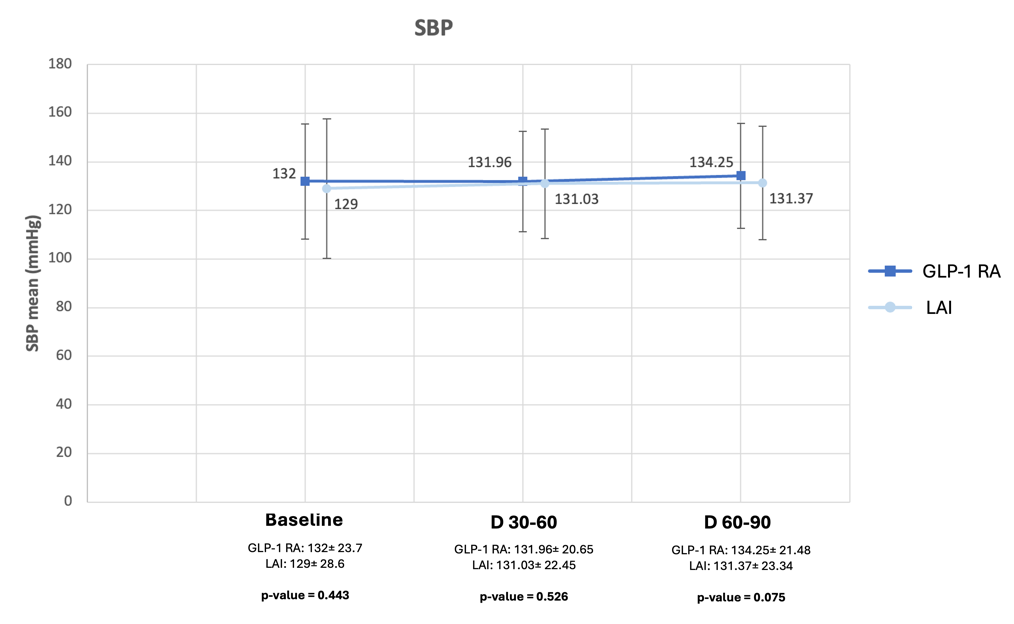
**

**Abbreviations:** GLP-1RAs, glucagon-like peptide receptor agonists; LAI, long‐acting insulin; SBP, systolic blood pressure. The p-values are indicated as follows: *p<0.05, **p<0.01. Graphical data are shown as mean ± SD.

**Suppl. Figure 6. Graphic abstract**
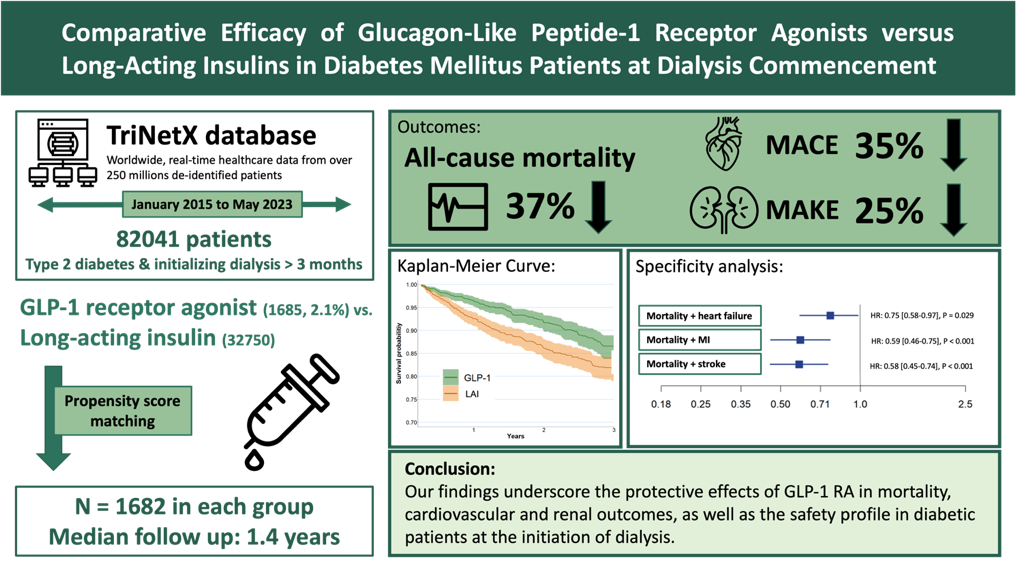


**Abbreviations:** GLP-1RAs, glucagon-like peptide receptor agonists; HR, hazard ratios; MACE, major adverse cardiac events; MAKE, major adverse kidney events

1. **STROBE statement checklist**

1. **Supplementary references**

1. Topaloglu U, Palchuk MB: **Using a Federated Network of Real-World Data to Optimize Clinical Trials Operations**. *JCO Clin Cancer Inform* 2018, **2**:1-10.

2. MacKenzie SL, Wyatt MC, Schuff R, Tenenbaum JD, Anderson N: **Practices and perspectives on building integrated data repositories: results from a 2010 CTSA survey**. *J Am Med Inform Assoc* 2012, **19**(e1):e119-124.

3. Hudson CL, Topaloglu U, Bian J, Hogan W, Kieber-Emmons T: **Automated Tools for Clinical Research Data Quality Control using NCI Common Data Elements**. *AMIA Jt Summits Transl Sci Proc* 2014, **2014**:60-69.

4. Kahn MG, Brown JS, Chun AT, Davidson BN, Meeker D, Ryan PB, Schilling LM, Weiskopf NG, Williams AE, Zozus MN: **Transparent reporting of data quality in distributed data networks**. *EGEMS (Wash DC)* 2015, **3**(1):1052.

5. Weiskopf NG, Hripcsak G, Swaminathan S, Weng C: **Defining and measuring completeness of electronic health records for secondary use**. *J Biomed Inform* 2013, **46**(5):830-836.
